# Cooperative effect of the VP1 amino acids 98E, 145A and 169F in the productive infection of mouse cell lines by enterovirus 71 (BS strain)

**DOI:** 10.1038/emi.2016.56

**Published:** 2016-06-22

**Authors:** Carla Bianca Luena Victorio, Yishi Xu, Qimei Ng, Tao Meng, Vincent TK Chow, Kaw Bing Chua

**Affiliations:** 1 Temasek Lifesciences Laboratory, National University of Singapore, Singapore 117604, Singapore; 2 Department of Microbiology and Immunology, Yong Loo Lin School of Medicine, National University of Singapore, Singapore 117545, Singapore

**Keywords:** EV71 adaptive mutations, molecular virology, enterovirus 71, mouse-cell adapted EV71, site-directed mutagenesis, RNA virus infectious clones, murine SCARB2 protein

## Abstract

Enterovirus 71 (EV71) is a neurotrophic virus that causes hand, foot and mouth disease (HFMD) and occasional neurological infection among children. It infects primate cells but not rodent cells, primarily due to the incompatibility between the virus and the expressed form of its receptor, scavenger receptor class B member 2 (SCARB2) protein, on rodent cells (mSCARB2). We previously generated adapted strains (EV71:TLLm and EV71:TLLmv) that were shown to productively infect primate and rodent cell lines and whose genomes exhibited a multitude of non-synonymous mutations compared with the EV71:BS parental virus. In this study, we aimed to identify mutations that are necessary for productive infection of murine cells by EV71:BS. Using reverse genetics and site-directed mutagenesis, we constructed EV71 infectious clones with specific mutations that generated amino acid substitutions in the capsid VP1 and VP2 proteins. We subsequently assessed the infection induced by clone-derived viruses (CDVs) in mouse embryonic fibroblast NIH/3T3 and murine neuroblastoma Neuro-2a cell lines. We found that the CDV:BS-VP1^K98E,E145A,L169F^ with three substitutions in the VP1 protein—K98E, E145A and L169F—productively infected both mouse cell lines for at least three passages of the virus in murine cells. Moreover, the virus gained the ability to utilize the mSCARB2 protein to infect murine cell lines. These results demonstrate that the three VP1 residues cooperate to effectively interact with the mSCARB2 protein on murine cells and permit the virus to infect murine cells. Gain-of-function studies similar to the present work provide valuable insight into the mutational trajectory required for EV71 to infect new host cells previously non-susceptible to infection.

## INTRODUCTION

Enterovirus 71 (EV71; species *Enterovirus A*; genus *Enterovirus*; family *Picornaviridae*)^1^ is a small non-enveloped RNA virus closely related to polioviruses. It is the major causative agent of hand, foot and mouth disease, an illness often associated with mild fever but that can also escalate to a severe disease with neurological involvement.^2, 3^ Cellular infection by EV71 can be divided into three major stages: cellular entry, expression of the viral proteins and hijacking of the host translational machinery, and production of viable viral progeny. Cellular entry, the first stage, is dependent on the EV71 capsid protein. The capsid is comprised of 60 identical units (protomers) assembled from four structural proteins (VP1–VP4).^4^ Interaction of the capsid with its cognate receptor on host cells triggers a series of events in viral uncoating: capsid expansion (to form the 135 S or A particle), extrusion of the VP1 N-terminus and VP4 from the capsid junction, which has a fivefold axis of symmetry, into the endosomal membrane and release of the viral genomic RNA into the host cell cytoplasm.^5, 6, 7, 8^ Once in the cytoplasm, the viral genome is translated via the cap-independent protein synthesis machinery of the host cell to generate 11 mature viral proteins that are required to shut off translation of the host proteins, inhibit cellular apoptosis during the early stages of infection, form an efficient viral genome replication complex, evade the host cell antiviral system and induce cell death in the later stages of infection.^9, 10, 11, 12, 13, 14, 15, 16^ Finally, after the viral genome has been sufficiently replicated and the viral structural proteins have been formed, the viral progeny are assembled. The four structural capsid proteins (VP1–VP4) self-assemble around the genomic RNA, and the virion undergoes a series of structural transitions to form mature, viable and infectious viral progeny.^17^

The main EV71 receptor that triggers viral uncoating has recently been identified as scavenger receptor class B member 2 (SCARB2).^18, 19^ Although the majority of EV71 strains recognize SCARB2 on primate cells,^20^ they are incompatible with SCARB2 proteins expressed on murine cells.^21^ The amino acid sequences of human and murine SCARB2 proteins exhibit only 84% identity and display significant structural divergence, especially in exon 4.^22, 23^ Cell lines derived from humans and non-human primates are highly susceptible to EV71 infection,^24^ but cell lines of rodent origin are not. Moreover, humans are the only known natural host and reservoir of EV71, and the virus does not infect other mammals. As an exception, rhesus and cynomolgus monkeys could be experimentally infected with EV71,^25, 26, 27, 28, 29, 30, 31, 32^ and suckling mice^33, 34, 35^ and gerbils (*Meriones unguiculatus*)^36, 37^ were susceptible to clinical isolates of the virus. Rodent cell lines that ectopically express the human SCARB2 (hSCARB2) protein, however, could be successfully infected with EV71.^22, 38^ Transgenic mice that express hSCARB2 also exhibited more severe signs of EV71-induced disease.^21, 39^ Thus, the incompatibility of EV71 with murine SCARB2 (mSCARB2) governs the restriction of EV71 infection of murine cells, and circumventing this incompatibility leads to an environment more conducive to infection.

In an attempt to overcome the natural incompatibility of EV71 and mSCARB2, our group adapted EV71 to grow in a cell line derived from mouse embryonic fibroblasts (NIH/3T3; CRL-1658). Through serial passage of a clinical isolate (EV71:BS) in this murine cell line, we generated 2 strains—EV71:TLLm (60 infection cycles) and EV71:TLLmv (100 infection cycles)—that productively infected a range of rodent cell lines.^40^ Previous viral RNA transfection studies have demonstrated that EV71:BS infection of murine cells is blocked at an early stage during cellular entry, and the adapted strains (EV71:TLLm and EV71:TLLmv) overcame this, presumably by adaptive mutations. Genome sequence comparison of the three strains revealed that EV71:TLLm and EV71:TLLmv accumulated multiple non-synonymous mutations in the capsid-encoding (P1) region, suggesting a possible correlation between the capsid mutations and the novel ability to infect rodent cell lines. In this study, we identified the capsid amino acid substitutions responsible for enabling EV71:BS to productively infect murine cell lines. By using reverse genetics and standard mutagenesis techniques, we synthesized plasmid cDNA clones of EV71:BS; incorporated site-specific mutations into the genome; and investigated the role of these mutations in productive infection of mouse cells.

## MATERIALS AND METHODS

### Plasmids, viruses, cell lines and primers

The plasmid pACYC177 (New England Biolabs, Ipswich, MA, USA), which is a low-copy plasmid (Supplementary Figure S1A), was used to generate the EV71 cDNA clones. The plasmid pCMV-T7pol, which expresses the phage T7 RNA polymerase, was used to produce the clone-derived viruses (CDVs).

Three strains of EV71 were used in this study. The clinical isolate EV71:BS (GenBank Accession NO KF514878) and the adapted strains EV71:TLLm (GenBank Accession NO KF514879.1) and EV71:TLLmv (GenBank Accession NO KF514880.1) have been previously described.^40^

African green monkey kidney Vero (CCL-81), mouse neuroblastoma Neuro-2a (CCL-131) and mouse embryonic fibroblast NIH/3T3 (CRL-1658) cells were purchased from the American Tissue Culture Collection (ATCC, Manassas, VA, USA). Cells were propagated and maintained as previously described.^40^

All primers used to construct the plasmid clones are listed in Supplementary Table S1. A detailed description of all the constructed plasmid complementary DNA DNA clones can be found in Supplementary Table S2. The primers and probes used in quantitative reverse transcription–PCR (qRT–PCR) are shown in Supplementary Table S3.

### Construction of the EV71 plasmid cDNA clones

We used a two-step cloning procedure to construct pACYC-BS, which is the pACYC plasmid containing the full-length cDNA of EV71:BS (Supplementary Figures S1B and S1C). To introduce specific mutations into pACYC-BS, we performed standard primer-mediated site-directed mutagenesis. The Supplementary Materials and Methods provides a full description of the procedures.

### Production of EV71 CDVs and assessment of the cellular infection phenotype

The constructed pACYC-BS clones were co-transfected with pCMV-T7pol into Vero cells using Lipofectamine 2000 reagent (Life Technologies, Carlsbad, CA, USA) following the manufacturer's protocol. Supernatants from transfected cells containing the CDVs were harvested 7–10 days after transfection, and viral titers were determined using the Reed and Muench method.^41^ CDVs were inoculated onto cells seeded overnight at an multiplicity of infection (MOI) of 1 by pre-adsorption, as previously described.^40^ We assessed the cellular infection phenotype by observing the progression of virus-induced lytic cytopathic effects (CPE) for several days post-inoculation (*p.i.*) and immunofluorescence staining of viral proteins as previously described.^40, 42^ Culture supernatants of infected cells were also harvested by centrifugation (1500 *g*; 10 min) and subjected to 10-fold serial dilutions with viral disaggregation using either chloroform or 1% sodium deoxycholate. Viral titers were determined using the Reed and Muench method.^41^

### Competitive viral entry and uncoating assays using recombinant soluble SCARB2 protein

These experiments were adapted from previously published procedures.^43^ We incubated the virus at an MOI of 100 with various concentrations of soluble mouse SCARB2 (mSCARB2) proteins (200 ng/μL stock solution) at 1000 ng, 500 ng, 250 ng and 0 ng for 2 h at 37 °C on a shaking platform. The virus was inoculated onto NIH/3T3 cells (6000 cells per well) seeded overnight and incubated at 37 °C. Cellular infection was assessed by observation of CPE and immunofluorescence detection of viral antigens at 48 h *p.i*.

For *in vitro* induction of viral uncoating, we incubated 10^6^ median cell culture infective doses (CCID_50_) of the virus with 200 ng soluble mSCARB2 protein at 4 °C on a shaking platform. The mixture was digested with 100 mg/mL RNase A (Qiagen, Hilden, Germany) for 10 min at room temperature (RT) to degrade susceptible RNA molecules, and samples were subsequently treated with 10 U of reaction RiboLock RNase inhibitor (Thermo Scientific, Waltham, MA, USA) for 10 min at RT to inactivate the RNases. Genomic RNA was extracted from intact viruses using an EZNA Viral RNA Kit (Omega Biotek, Norcross, GA, USA) following the manufacturer's protocol, and eluted RNA samples were stored in −80 °C until further use. In similar experiments, virus at an MOI of 10 was incubated with various concentrations of mSCARB2 protein (25, 50, 100 and 200 ng) or 200 ng BSA as a non-specific protein (NSP) control at 4 °C overnight. The treated virus was inoculated onto seeded NIH/3T3 cells (10^5^ cells per well) for 1 h at 4 °C, and then cells were washed 3 × with sterile, cold PBS and incubated in DMEM (1% FBS) for 2 h at 37 °C. Total cellular RNA was extracted from the inoculated cells using an AxyPrep Multisource Total RNA Miniprep kit (Axygen, Union City, CA, USA) following the manufacturer's protocol, and eluted RNA samples were stored in −80 °C until further use. The Supplementary Materials and Methods describe the procedures used in the recombinant expression of soluble SCARB2 proteins.

### Blocking viral cellular entry using anti-mSCARB2 rabbit sera

These experiments were adapted from previously published procedures.^43^ NIH/3T3 cells seeded overnight in 96-well plates (1 × 10^4^ cells per well) were incubated with twofold serial dilutions (1:20 to 1:640) of anti-mSCARB2 rabbit sera for 1 h at 37 °C. Cells were subsequently inoculated with virus (100 MOI) for 1 h at 37 °C. Cells were washed 2 × in PBS and incubated in DMEM (1% FBS) for 1 h at 37 °C. Cellular infection was assessed by detection of CPE and measurement of viral titer in cell culture supernatants harvested three days *p.i*.

In another set of experiments, NIH/3T3 cells were seeded in 12-well plates (10^5^ cells per well) and incubated with various concentrations (1, 2, 4 or 8 μg) of anti-mSCARB2 rabbit serum in a rocking platform at 4 °C for 1 h. Alternatively, cells were treated with rabbit serum against Saffold virus L protein (2 μg) as a control serum against a NSP. Treated cells were subsequently washed with cold, sterile PBS 3 × and inoculated with virus at an MOI of 10 at 4 °C for 1 h on a rocking platform. Cells were then washed 3 × in cold PBS and incubated with DMEM (1% FBS) at 37 °C for various durations (1–4 h). Total cellular RNA was extracted using the AxyPrep Multisource Total RNA Miniprep kit following the manufacturer's protocol, and eluted RNA samples were stored in −80 °C until further use.

### QRT–PCR of collected RNA samples

RNA samples were quantified using a One-Step RT-PCR kit and a Rotor-Gene Q machine (Qiagen) following the manufacturer's protocols. Primers and gene-specific probes targeting a conserved region of the EV71 5′ untranslated region (EV5′NC) were used to detect the virus, while primers and probes targeting an exon of human β-actin were used as internal controls. Primer and probe sequences are listed in Supplementary Table S3. Absolute quantitation using established standardized curves was performed for RNA samples extracted in the *in vitro* uncoating studies. Relative quantitation using the ΔΔC_T_ method^44, 45^ was performed to measure viral RNA from total cellular RNA samples using β-actin as an endogenous control.

### Animal infections

Procedures for handling and infection of mice were approved by the Institutional Animal Care and Use Committee of Temasek Lifesciences Laboratory (TLL-IACUC Approval NO 14/023), which follows the guidelines specified by the National Advisory Committee on Laboratory Animal Research (NACLAR) of Singapore. Groups of eight 6-day-old BALB/c pups were inoculated via the intraperitoneal (I.P.) route with 10^6^ CCID_50_ of virus at day 0. Mock-infected mice were inoculated with equal volumes of DMEM (1% FBS). Inoculated mice were observed twice daily for signs of infection, and body weights were measured once daily. The general criteria for euthanasia followed previously established guidelines^35^ and included (i) loss of >20% maximum recorded body weight, (ii) paralysis persisting >48 h, (iii) absence of feeding or inability to feed, (iv) inability to self-right, and (v) altered state of consciousness presenting as either stupor or coma. On appearance of these disease manifestations, mice were killed by I.P. injection of pentobarbitone (100 mg/kg). Animals that survived the 28-day observation period were also killed by pentobarbitone. Blood samples were terminally collected by cardiac puncture for subsequent serum analysis for neutralizing antibodies. Serum neutralization tests are described in the Supplementary Materials and Methods.

### Statistical analysis

All graphs and statistical analyses were performed using GraphPad Prism ver. 6.01 for Windows (GraphPad Software, La Jolla, CA, USA, www.graphpad.com). When choosing the appropriate statistical tests, the normal distribution of data was first confirmed with QQ plots and Kolmogorov–Smirnov tests. Comparisons of mean values for normally distributed data with unequal variance were performed using *t-*tests with Welch's correction, while comparisons of medians for non-normally distributed data were performed with Mann–Whitney *U*-tests.

## RESULTS

### Confirmation that the EV71 capsid protein restricts murine cell infection

Our previous studies demonstrated that transfection of viral genomic RNA into cells leads to induction of CPE, expression of viral proteins and generation of viable viral progeny.^40^ Transfection of genomic RNA from EV71:BS, EV71:TLLm or EV71:TLLmv into Vero, NIH/3T3 and Neuro-2a cells (Supplementary Figure S2A) induced significant CPE and resulted in the expression of viral proteins (Supplementary Figures S2B and S2C). Passage of EV71:TLLm and EV71:TLLmv transfection supernatants from Vero, NIH/3T3 or Neuro-2a cells onto fresh Vero and NIH/3T3 cells also resulted in significant CPE (Supplementary Figures S2D and S2E), confirming the presence of viable viral progeny in transfection supernatants. Moreover, passage of EV71:BS RNA transfection supernatants from Vero, NIH/3T3 or Neuro-2a cells onto fresh Vero, but not NIH/3T3, cells resulted in CPE and expression of viral proteins (Supplementary Figures S2F and S2G). These results demonstrate that the transfected cells are permissive to infection. They allow viral replication and contain the host proteins required for completion of the EV71 infection cycle. Once the viral RNA is inside the cell, the host cellular machinery expresses the viral proteins required for the replication of the viral genome and production of viral progeny. This was observed even in cell lines previously determined to be non-susceptible to EV71:BS infection (NIH/3T3 and Neuro-2a cells).^40^ These data suggest that the restriction of EV71:BS infection of murine cells is due to a defect in cellular entry, a process predominantly attributed to the viral capsid protein.

**Figure 1:**
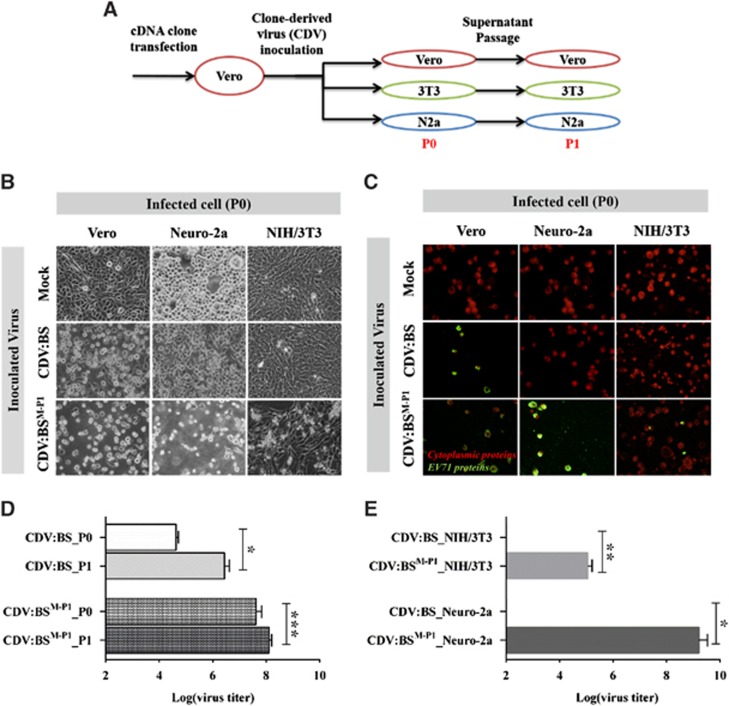
Enabling EV71 infection of mouse cell lines by replacing the EV71:BS capsid with EV71:TLLm capsid sequences. (**A**) Schematic representation of the cell infection experiments. *3T3*, NIH/3T3; *N2a*, Neuro-2a; *P0*, passage 0; *P1*, passage 1. (**B–E**) Assessment of cellular infection induced by CDVs at 48 h *p.i.* Cells inoculated with CDVs at an MOI of 1 were observed for evidence of lytic CPE (**B**) and expression of viral proteins (**C**). Production of viable viral progeny was also determined by measuring viral titers from inoculated Vero (**D**), NIH/3T3 and Neuro-2a cells (**E**). Live cell images were taken at × 40 magnification and are shown in grayscale. Fluorescence images were taken at × 40 magnification and are shown with enhanced contrast. Viral antigens were labeled with FITC (green), and cells were counterstained with Evans blue (red). Titers from Vero cells were measured at P0 and P1, while titers from NIH/3T3 and Neuro-2a cells were measured at P0. Titrations were performed with dilutions of 10^−1^ to 10^−10^ in Vero cells following viral disaggregation with chloroform. The results are representative of two independent studies. For **D** and **E**, a modified *t-*test with Welch's correction for unequal variance was used to compare mean values (*n*=4). Error bars represent the s.d. *P<0.05; **P<0.005, ***P<0.0005. clone-derived virus, CDV; cytopathic effect, CPE; multiplicity of infection, MOI.

To confirm the role of the capsid protein in restricting murine cell infection by EV71:BS, we replaced the capsid-encoding genes (P1 region) of pACYC-BS, which is the plasmid containing the full-length cDNA of EV71:BS, with equivalent sequences from EV71:TLLm to produce a chimeric plasmid pACYC-BS^ΔM-P1^ (Supplementary Table S2; Supplementary Figures S1B and S1C). We transfected the pACYC-BS and pACYC-BS^ΔM-P1^ plasmids separately into Vero cells and harvested the supernatants at seven days after transfection to obtain the CDVs CDV:BS and CDV:BS^M-P1^, respectively. CDV:BS^M-P1^ expresses the capsid protein of EV71:TLLm and the non-structural proteins of EV71:BS, while CDV:BS expresses all proteins of EV71:BS. The generated CDVs were subsequently inoculated into Vero, NIH/3T3 and Neuro-2a cell cultures at an MOI of 1 to assess the phenotype of cellular infection. Infection supernatants were further passaged in the same cell line to ascertain production of viable viral progeny (Figure 1A).

CDV:BS and CDV:BS^M-P1^ induced full CPE and viral protein expression in inoculated Vero cells. In contrast, only CDV:BS^M-P1^ induced significant CPE and viral antigen production in NIH/3T3 and Neuro-2a cells (Figures 1B and C). Viable viral progeny were also detected in Vero cells inoculated with either CDV, as determined by viral titer measurements in passages 0 and 1 (P0 and P1). We observed an increase in titers from P0 to P1 in both CDV:BS (*P*=0.0055) and CDV:BS^M-P1^ (*P*=0.0005) (Figure 1D), confirming that the constructed clones are infectious. In inoculated murine cells, however, only CDV:BS^MP1^ resulted in detectable viral titers in P0. Compared with CDV:BS, elevated titers were measured in the NIH/3T3 (*P*=0.0027) and Neuro-2a (*P*=0.0051) cells inoculated with CDV:BS^M-P1^ (Figure 1E). These results demonstrate that CDV:BS, similar to EV71:BS, infected primate cells but not murine cells. In contrast, CDV:BS^M-P1^ productively infected both primate and murine cells, similar to EV71:TLLm and EV71:TLLmv. These experiments confirm that the capsid restricts EV71:BS infection of murine cells at the stage of cellular entry, and replacing the capsid protein with the EV71:TLLm capsid sequence permits productive infection of mouse cells. In addition, the results also suggest that the molecular determinants that enable EV71:TLLm (and EV71:TLLmv) to productively infect murine cells reside within the capsid protein.

### Identification of determinants in the capsid that enable entry of EV71:BS into mouse cell lines

We previously determined that the majority of differences in the amino acid sequence between the aligned polyproteins of EV71:BS and the adapted strains (either EV71:TLLm or EV71:TLLmv) were located in the capsid-encoding (P1) region.^40^ To determine which of these could facilitate entry into murine cells, we selected several candidates based on the assumption that these molecular determinants should be (i) common to both EV71:TLLm and EV71:TLLmv and (ii) either surface-exposed or located near the capsid canyon region to potentially interact with the viral receptor on host cells. We therefore focused on five amino acid substitutions in VP1 and VP2 that are found in both adapted strains. VP1-K98E and E145A were selected because of their surface exposure, and L169F was selected because of its proximity to the canyon region^46^ that participates in binding to the viral receptor (Supplementary Figure S3). Similarly, the VP2 substitutions S144T and K149I were selected because they are in the surface-exposed EF loop or puff region that forms the floor of the canyon and are located within previously identified neutralizing epitopes^47, 48^ (Supplementary Figure S4).

**Figure 2:**
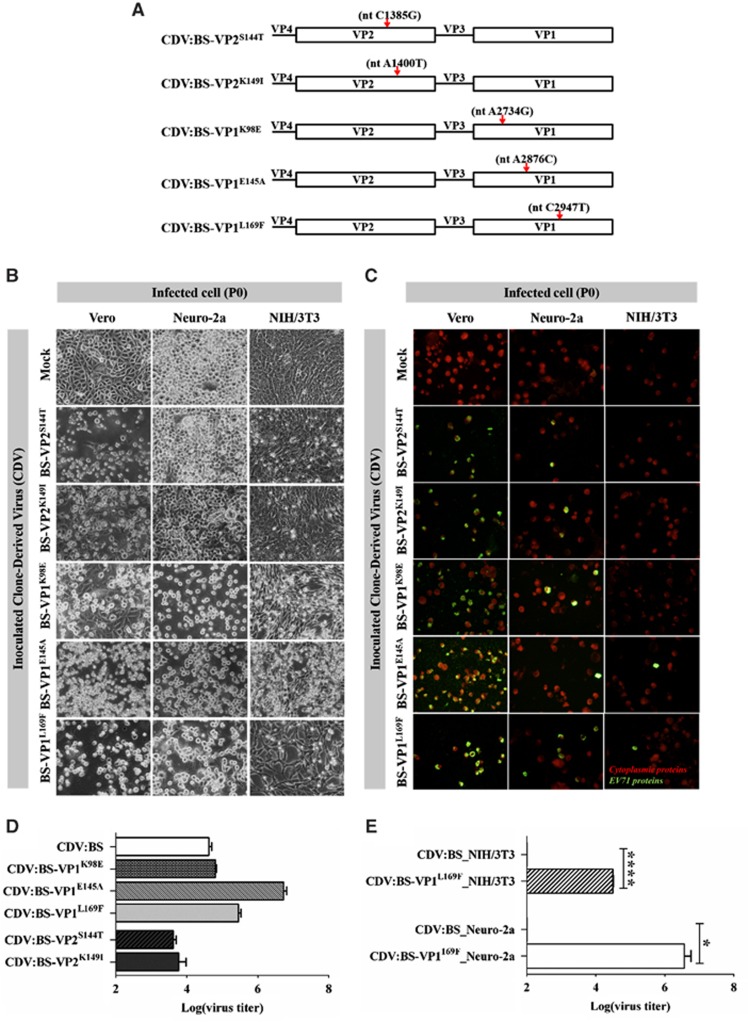
Cell infection induced by CDVs with single amino acid substitutions in the VP1 or VP2 proteins of EV71:BS. (**A**) Schematic map depicting the location of the nucleotide (nt) mutations introduced into the genomes of various CDVs. (**B**–**E**) Cellular infection in Vero, NIH/3T3 and Neuro-2a cells inoculated with CDVs at an MOI of 1 was assessed by detecting virus-induced CPE (**B**) and viral antigens (**C**) at 72 h *p.i.* at passage 0 (P0). Live cell images were taken at × 40 magnification and are shown in grayscale. Fluorescence images were taken at × 40 magnification and are shown with enhanced contrast. Viral antigens were labeled with FITC (green), and cells were counterstained with Evans blue (red). Viral titers were also measured from Vero cells inoculated with CDVs (**D**) and murine cells (NIH/3T3 and Neuro-2a) inoculated with CDV:BS-VP1^L169F^ (**E**). The results are representative of two independent experiments. For **D** and **E**, a modified *t-*test with Welch's correction for unequal variance was used to compare mean values (*n*=4). Error bars represent the s.d.. *P<0.05, ****P<0.0001. clone-derived virus, CDV; cytopathic effect, CPE; multiplicity of infection, MOI.

To determine which of these substitutions enables EV71:BS to enter mouse cell lines, we incorporated individual mutations into the pACYC-BS plasmid by site-directed mutagenesis (Figure 2A and Supplementary Table S2). All CDVs induced full CPE and viral protein expression in inoculated Vero cells. They also induced viral protein expression in Neuro-2a cells, but only CDVs with VP1 substitutions elicited full CPE (Figures 2B and C). In contrast, only CDVs with the VP1 substitutions induced CPE in NIH/3T3 cells, and viral antigens were observed only in cells inoculated with CDV:BS-VP1^L169F^ and CDV:BS-VP1^E145A^. Moreover, supernatants derived from Vero cells inoculated with any of the CDVs had high titer values (Figure 2D), confirming that the clones are infectious. In Neuro-2a and NIH/3T3 cells, however, only supernatants derived from cells inoculated with CDV:BS-VP1^L169F^ had detectable titer values. Elevated CDV:BS-VP1^L169F^ titers were measured in NIH/3T3 (*P*<0.0001) and Neuro-2a cells (*P*=0.0391) compared with the respective CDV:BS titers (Figure 2E). These results demonstrate that CDVs with single amino acid substitutions in the VP1 (K98E, E145A and L169F) or VP2 (S144T and K149I) proteins of EV71:BS were able to enter murine cells and express viral proteins. Thus, all of these substitutions enable entry of EV71:BS into murine cells. However, only CDV:BS-VP1^L169F^ was able to productively infect murine cells; it entered, replicated and produced viable viral progeny in NIH/3T3 and Neuro-2a cells.

### Identification of determinants that enable EV71:BS to productively infect mouse cell lines

**Figure 3:**
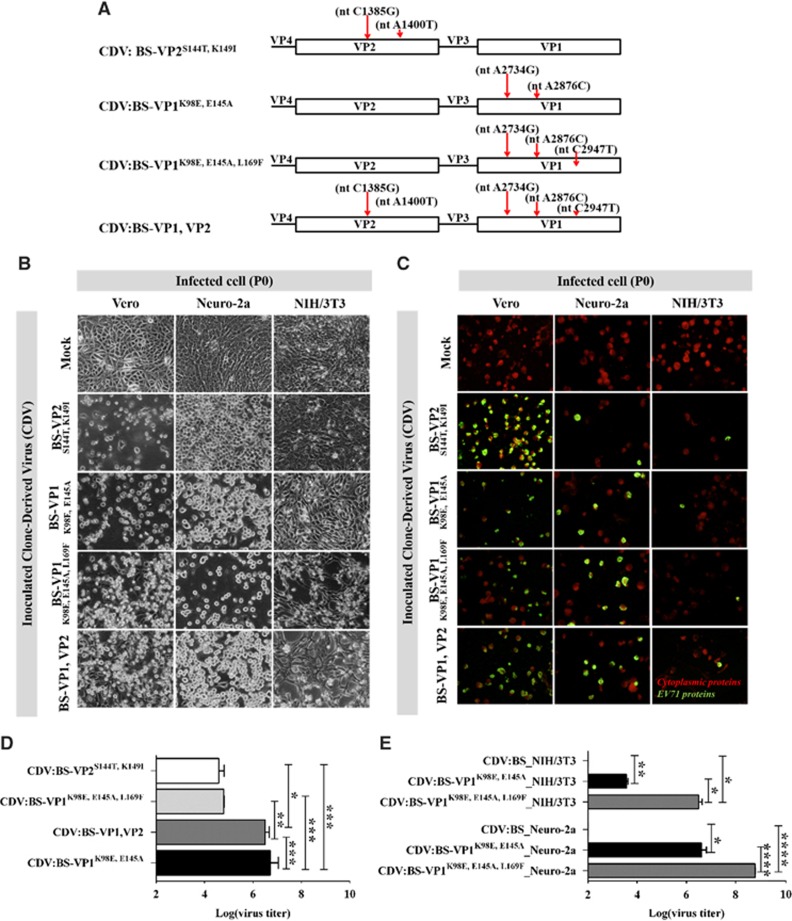
Cell infection induced by CDVs with multiple amino acid substitutions in the VP1 and VP2 proteins of EV71:BS. (**A**) Schematic map depicting the location of the nucleotide (nt) mutations introduced into the genomes of various CDVs. (**B**–**E**) Cellular infection in Vero, NIH/3T3 and Neuro-2a cells inoculated with CDVs at an MOI of 1 was assessed by detecting virus-induced CPE (**B**) and viral antigens (**C**) at 72 h p.i. at passage 0 (P0). Live cell images were taken at × 40 magnification and are shown in grayscale. Fluorescence images were taken at × 40 magnification and are shown with enhanced contrast. Viral antigens were labeled with FITC (green), and cells were counterstained with Evans blue (red). Viral titers were also measured from Vero cells inoculated with all CDVs (**D**) and murine cells (NIH/3T3 and Neuro-2a) inoculated with CDV:BS-VP1^K98E,E145A^ or CDV:BS-VP1^K98E,E145A,L169F^ (**E**). Titers from all cells were measured at passage 0 (P0). Titrations were performed with dilutions of 10^−1^ to 10^−10^ in Vero cells following viral disaggregation with chloroform. The results are representative of two independent experiments. For **D** and **E**, a modified *t-*test with Welch's correction for unequal variance was used to compare mean values (*n*=4). Error bars represent the SD *P<0.05, **P<0.005, ***P<0.0005, ****P<0.0001. clone-derived virus, CDV; cytopathic effect, CPE; multiplicity of infection, MOI.

We also introduced various combinations of the VP1 and VP2 substitutions into the pACYC-BS plasmid by site-directed mutagenesis (Figure 3A and Supplementary Table S2). Several of the CDVs induced significant CPE, while all CDVs expressed viral proteins in Vero, NIH/3T3 and Neuro-2a cells (Figures 3B and 3C). High titers were also recorded in the supernatants derived from Vero cells, confirming that the clones were infectious. The highest titers were observed in the CDV:BS-VP1^K98E,E145A^ and CDV:BS-VP1 and VP2-inoculated cells (Figure 3D). In contrast, only CDV:BS-VP1^K98E,E145A^ and CDV:BS-VP1^K98E,E145A,L169F^ resulted in high viral titer values in NIH/3T3 and Neuro-2a cells. Compared with CDV:BS, higher CDV:BS-VP1^K98E,E145A^ titer values were recorded in NIH/3T3 (*P*<0.0011) and Neuro-2a cells (*P*=0.039) (Figure 3E). Similar results were observed for CDV:BS-VP1^K98E,E145A,L169F^ (NIH/3T3, *P*=0.0136; Neuro-2a cells, *P*<0.0001). These findings demonstrate that simultaneous introduction of VP1-K98E and E145A into EV71:BS leads to productive infection of NIH/3T3 and Neuro-2a cells. In contrast to CDV:BS-VP1^K98E^ or CDV:BS-VP1^E145A^, CDV:BS-VP1^K98E,E145A^ not only entered and replicated in the infected cells, but it also produced viable viral progeny. Furthermore, combining VP1-K98E, E145A and L169F together enhanced the infectivity of the resulting CDV in murine cells. In NIH/3T3 cells, higher titers were observed for CDV:BS-VP1^K98E,E145A,L169F^ compared with CDV:BS-VP1^K98E,E145A^-inoculated cells (*P*=0.0136) (Figure 3E). Similar results were observed in Neuro-2a cells (*P*<0.0001). These findings suggest that the three amino acid residues in VP1 (98E, 145A and 169F) may constitute the minimum determinants for mouse cell adaptation necessary to permit productive infection of EV71:BS in murine cell lines.

### Stability of the mouse cellular infection phenotype and genomic stability of the VP1 substitutions K98E, E145A and L169F

**Figure 4:**
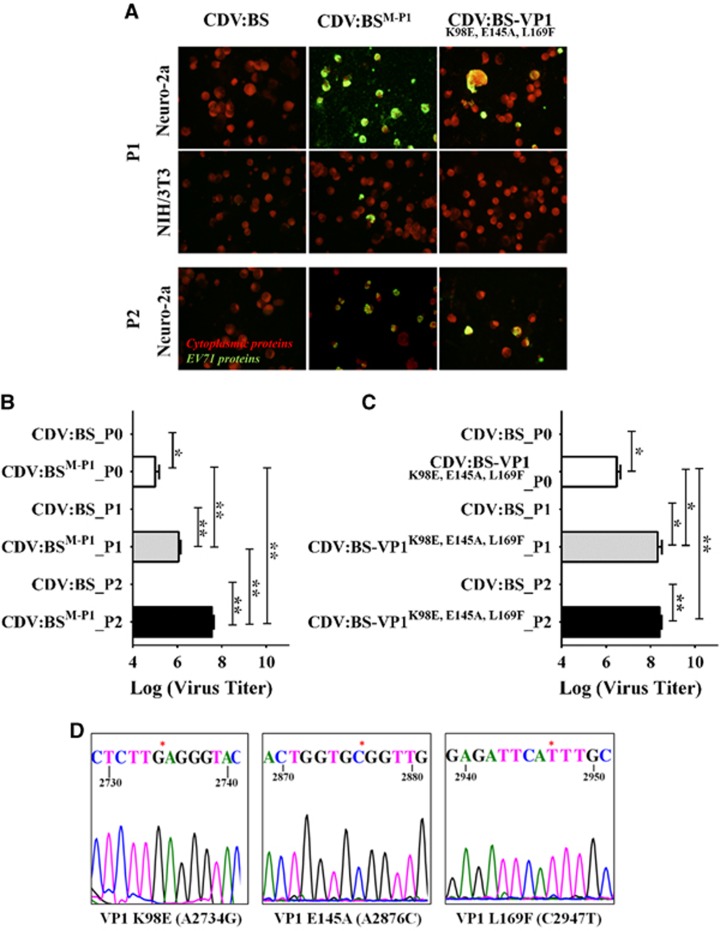
Phenotypic and genotypic stability of CDV:BS^M-P1^ and CDV:BS-VP1^K98E,E145A,L169F^ in the context of mouse cellular infection. (**A**) Immunofluorescence detection of viral antigens in NIH/3T3 and Neuro-2a cells inoculated with CDV at an MOI of 1 at 72 h p.i. CDV:BS^M-P1^ and CDV:BS-VP1^K98E,E145A,L169F^ infection supernatants were passaged successively for three infection cycles in Neuro-2a and NIH/3T3 cells, and infection was assayed at P1 (passage 1) and P2 (passage 2). Fluorescence images were taken at × 40 magnification and are shown with enhanced contrast. Viral antigens were labeled with FITC (green), and cells were counterstained with Evans blue (red). (**B, C**) Viral titers in infected lysates of Neuro-2a cells inoculated with either CDV:BS^M-P1^ (**B**) or CDV:BS-VP1^K98E,E145A,L169F^ (**C**). Titers were measured at P0, P1 and P2. Viral titrations were performed with dilutions of 10^−3^ to 10^−10^ in Vero cells following viral disaggregation with 1% sodium deoxycholate. (**D**) Representative sequence of regions of the VP1 protein of CDV:BS-VP1^K98E,E145A,L169F^ determined at P2 in Neuro-2a cells. The mutation sites are marked with an asterisk. The results are representative of two independent experiments. For **B** and **C**, a modified *t-*test with Welch's correction for unequal variance was used to compare mean values (*n*=4). Error bars represent the SD *P<0.05, **P<0.005. clone-derived virus, CDV.

We identified four mutant CDVs that productively infect NIH/3T3 and Neuro-2a cells—CDV:BS^M-P1^, CDV:BS-VP1^L169F^, CDV:BS-VP1^K98E,E145A^ and CDV:BS-VP1^K98E,E145A,L169F^. To evaluate whether these CDVs induce stable productive infections of murine cells, we performed three viral passages at an MOI of 1 in NIH/3T3 and Neuro-2a cells to simulate a genetic bottleneck. Of the four CDVs, only CDV:BS^M-P1^ and CDV:BS-VP1^K98E,E145A,L169F^ exhibited sustainable productive infection of Neuro-2a cells for three cycles and of NIH/3T3 cells for two cycles. Viral antigens were detected in Neuro-2a cells at both P1 and P2, while antigens were observed in NIH/3T3 cells only at P1 (Figure 4A). Compared with CDV:BS, higher titers were recorded for Neuro-2a cells inoculated with either CDV:BS^M-P1^ or CDV:BS-VP1^K98E,E145A,L169F^ at P0, P1 and P2. In addition, higher titers were determined relative to the previous passage at each infection cycle. For CDV:BS^M-P1^, titers at P1 were higher than the P0 titers (*P*=0.0038), and the P2 titers were higher than P1 (*P*=0.0031) (Figure 4B). For CDV:BS-VP1^K98E,E145A,L169F^, the P2 and P1 titers were higher than the P0 titers (P1 vs P0, *P*=0.0376; P2 vs P0, *P*=0.0032) (Figure 4C).

To confirm the genomic stability of the three VP1 substitutions, we sequenced the VP1 gene region of the virus derived from passage P2 in Neuro-2a cells. The introduced nucleotide mutations A2734G, A2876C and C2947T resulting in amino acid substitutions VP1-K98E, E145A and L169F, respectively, were evident in CDV:BS-VP1^K98E,E145A,L169F^ (Figure 4D) and CDV:BS^M-P1^ (data not shown). These findings demonstrate that the infection phenotypes induced by CDV:BS-VP1^K98E,E145A,L169F^ and CDV:BS^M-P1^ are sustainable in Neuro-2a cells for at least three passages and would induce sustained infection in these cells. Moreover, the VP1 mutations are stable in the viral genome after the third passage, suggesting that they may persist even in later passages. In contrast, both CDVs did not achieve sustainable infection in NIH/3T3 cells, and instead the population collapsed as expected for populations with unestablished fitness subjected to a genetic bottleneck.^49^ This suggests that further genetic alterations are necessary to establish stable infection in mouse fibroblast cells, as reflected in the multitude of non-synonymous mutations observed in the non-structural proteins of both EV71:TLLm and EV71:TLLmv.^40^

### Assessing the role of the SCARB2 protein in the cellular infection of adapted EV71 strains

**Figure 5:**
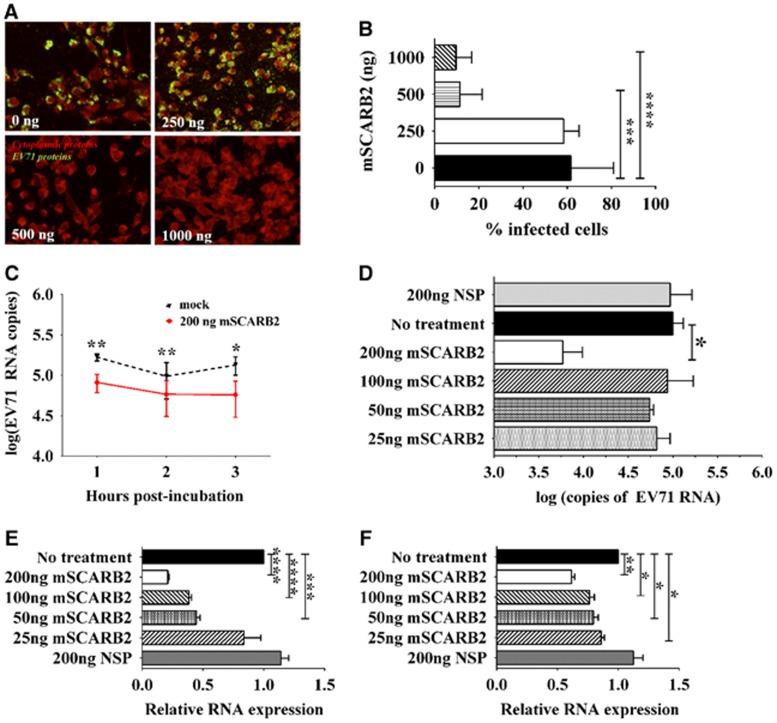
Viral competitive entry/uncoating assays by pre-incubation of EV71 with the murine SCARB2 (mSCARB2) protein. (**A, B**) Cellular infection of NIH/3T3 cells with EV71:TLLmv pre-incubated with various concentrations of soluble mSCARB2 protein prior to viral inoculation. Viral antigens were detected by fluorescence immunostaining at 48 h p.i. (**A**). Fluorescence images were taken at × 40 magnification and are shown with enhanced contrast. Viral antigens were labeled with FITC (green), and cells were counterstained with Evans blue (red). The number of infected cells was counted in ten independent fields of view (**B**). (**C**, **D**) Viral uncoating study, where 10^6^ CCID_50_ of EV71:TLLmv was incubated with various amounts of soluble mSCARB2 protein. Viral RNA copies were absolutely quantified after incubation with 200 ng of mSCARB2 for 3 h (**C**) or after incubation with various concentrations of mSCARB2 for 2 h (**D**). (**E**, **F**) Effect of virus pre-incubation with soluble mSCARB2 on cellular infectivity. Relative quantitation of EV71 RNA in total cellular RNA extracted from NIH/3T3 cells inoculated with either EV71:TLLmv (**E**) or EV71:TLLm (**F**) pre-incubated with soluble mSCARB2 at an MOI of 10. Total cellular RNA was extracted at 2 h *p.i.*, and viral RNA was quantified by the ΔΔC_T_ method using β-actin as an internal control. For **B**, a Mann–Whitney *U*-test was used to compare medians (*n*=8). Error bars represent the range. For **C–F**, a *t-*test with Welch's correction for unequal variance was used to compare mean values (*n*=4). Error bars represent the s.d. *P<0.05, **P<0.005, ***P<0.0005, ****P<0.0001. cell culture infective dose, CCID; non-specific protein, NSP.

EV71 utilizes SCARB2 as its uncoating receptor for host cell infection.^18, 19^ To determine whether EV71:TLLm and EV71:TLLmv also utilize SCARB2 to infect murine cells, we performed several assays. In the viral competitive entry experiment, we pre-incubated EV71:TLLmv with various concentrations of soluble mSCARB2 protein prior to inoculation onto NIH/3T3 cells. We assessed viral infectivity by immunostaining for viral antigens in cells at 48 h *p.i.* and counting the number of infected cells. Pre-incubation of the virus with soluble mSCARB2 reduced viral infectivity in a dose-dependent manner, and fewer viral antigens were detected in cells inoculated with virus pre-incubated with 500 ng or 1 μg soluble mSCARB2 (Figure 5A). Compared with cells inoculated with mock-incubated EV71:TLLmv, lower percentages of infection were recorded in cells inoculated with virus pre-incubated with 500 ng (*P*=0.0004) and 1 μg (*P*<0.0001) of soluble mSCARB2 (Figure 5B).

In viral uncoating studies, we quantified the amount of EV71 RNA extracted from intact viruses after *in vitro* incubation with mSCARB2. Compared with mock-incubated virus, lower amounts of viral RNA were measured in samples pre-incubated with 200 ng of mSCARB2 at 1 h (*P*=0.0009), 2 h (*P*=0.0036) and 3 h (*P*=0.0141) *p.i.* (Figure 5C). Comparing the effect of various concentrations of mSCARB2 at 2 h post-incubation with virus, we observed a similar reduction in viral RNA quantity in samples incubated with 200 ng of protein (*P*=0.0084) but not in samples incubated with the lower doses of mSCARB2 or with a NSP (Figure 5D). Furthermore, we inoculated the pre-incubated virus onto NIH/3T3 cells and quantified the viral RNA from extracted total cellular RNA. Compared with mock-incubated controls, lower amounts of EV71 RNA were detected in samples inoculated with EV71:TLLmv pre-incubated with 50 ng (*P*=0.0003), 100 ng (*P*<0.0001), or 200 ng (*P*<0.0001) of mSCARB2 protein (Figure 5E). In addition, pre-incubation of EV71:TLLm with 25 ng (*P*=0.0157), 50 ng (*P*=0.0142), 100 ng (*P*=0.0096) or 200 ng (*P*=0.0006) of mSCARB2 resulted in a similar reduction in EV71:TLLm RNA detected in inoculated cells (Figure 5F). This reduction was not observed in cells inoculated with virus pre-incubated with 200 ng of NSP. Similar results were also obtained when EV71:TLLmv was pre-incubated with soluble hSCARB2 protein (Supplementary Figure S5). In contrast, pre-incubation of EV71:BS with hSCARB2 resulted in a reduction in the amount of viral RNA, but mSCARB2 had no such effect (Supplementary Figure S6).

**Figure 6:**
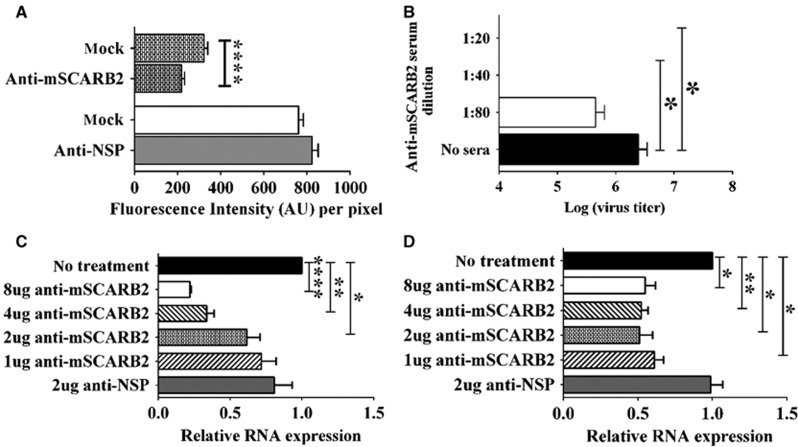
Viral cellular entry-blocking assays by pre-incubation of NIH/3T3 cells with serum against the mSCARB2 protein. (**A**) Quantitation of fluorescence intensity in FITC-labeled EV71 capsid proteins from virus bound to cells. NIH/3T3 cells were either mock-incubated or pre-incubated with anti-mSCARB2 rabbit serum prior to inoculation with EV71:TLLmv for 1 h. The other panel compares the fluorescence intensity between mock-treated cells and cells pre-incubated with serum against a nonspecific protein (NSP). Quantitation was performed in 100 independent fields of view. (**B**) Viral titers at three days *p.i.* in infected cells pre-incubated with various dilutions of anti-mSCARB2 sera. Titrations were performed in NIH/3T3 cells (10^−3^ to 10^−10^ dilutions) following viral disaggregation using 1% sodium deoxycholate (*n*=8). (**C, D**) Relative quantitation of viral RNA from total RNA extracted from cells pre-incubated with various amounts of anti-mSCARB2 sera prior to inoculation with either EV71:TLLmv (**C**) or EV71:TLLm (**D**) (*n*=4). Total cellular RNA was extracted at 2 h *p.i.*, and viral RNA was quantified by the ΔΔC_T_ method using β-actin as an internal control. For **A–D**, a *t-*test with Welch's correction for unequal variance was used to compare mean values. Error bars represent the s.d. *P<0.05, **P<0.005, ****P<0.0001.

In another set of experiments, we blocked cellular entry of the virus by pre-incubating NIH/3T3 cells with anti-mSCARB2 rabbit serum, which contains polyclonal antibodies that recognize both recombinant soluble SCARB2 proteins expressed in *Escherichia coli* and native SCARB2 proteins from mammalian cell lysates (Supplementary Figure S7), prior to inoculation with EV71:TLLmv at an MOI of 100. As a control, we pre-incubated cells with rabbit serum against a NSP. Subsequently, we measured the amount of virus bound to the cells by quantifying the immunofluorescence signals obtained after immunostaining for EV71 capsid proteins. Compared with mock-treated controls, cells incubated with anti-mSCARB2 sera exhibited a reduction in fluorescence signals (*P*<0.0001), which was not observed in cells pre-incubated with anti-NSP sera (Figure 6A).

In a similar experiment, we inoculated EV71:TLLmv onto NIH/3T3 cells pre-incubated with various dilutions of anti-mSCARB2 sera and assessed cellular infection by measuring viral titers at 3 days *p.i.* and quantifying the amount of viral RNA in cells at 2 h p.i. Pre-incubation of cells with low dilutions of mSCARB2 antiserum (1:20 to 1:40) resulted in a dose-dependent reduction of viral titers compared with mock-incubated controls (Figure 6B). Moreover, pre-incubation of NIH/3T3 cells with 8 μg (*P*<0.0001), 4 μg (*P*=0.0010) or 2 μg (*P*=0.0257) of anti-mSCARB2 serum resulted in lower amounts of EV71:TLLmv RNA detected in inoculated cells, which was not observed in cells pre-incubated with anti-NSP sera (Figure 6C). Similar results were obtained when treated cells were inoculated with EV71:TLLm (8 μg, *P*=0.0072; 4 μg, *P*=0.0019; 2 μg, *P*=0.0111; 1 μg, *P*=0.0079) (Figure 6D).

All these results demonstrate that the interaction between EV71:TLLmv (or EV71:TLLm) and mSCARB2 is crucial for the infection of murine cells. Blocking this interaction by incubating cells with anti-mSCARB2 sera reduced cellular infection. Similarly, virus pre-incubation with soluble mSCARB2 led to a reduction in viral infectivity, suggesting that the interaction of the virus with mSCARB2 resulted in either viral sequestration or alteration of the capsid structure to effectively disable it from infecting murine cells. Thus, we confirmed that EV71:TLLmv and EV71:TLLm use mSCARB2 to infect murine cell lines.

### Assessing the role of SCARB2 in infection of CDV:BS^M-P1^ and CDV:BS-VP1^K98E,E145A,L169F^ in mouse cell lines

**Figure 7:**
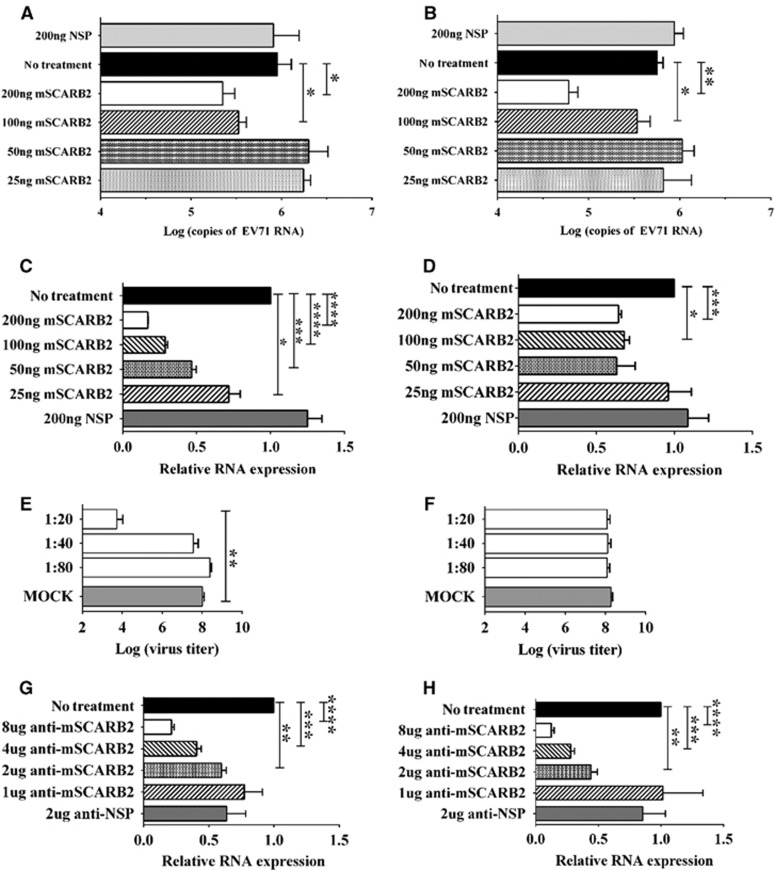
Assessing the role of the mSCARB2 protein in CDV:BS^M-P1^ and CDV:BS-VP1^K98E,E145A,L169F^ infection of murine cells. (**A**–**D**) Pre-incubation of 10^6^ CCID_50_ CDVs with the mSCARB2 protein for *in vitro* uncoating (**A, B**) or for cellular infection studies of NIH/3T3 cells (**C**, **D**). The viral RNA in the samples was extracted and quantified by RT–PCR (qRT–PCR). (**E–H**) Blocking viral entry by incubating NIH/3T3 cells with anti-mSCARB2 sera prior to inoculation with virus at an MOI of 10. Infection was assessed by determining viral titers in culture supernatant with dilutions of 10^-1^ to 10^−10^ in Vero cells at three days *p.i.* following chloroform viral disaggregation (**E**, **F**), and relative quantitation of EV71 RNA in extracted total cellular RNA by the ΔΔC_T_ method using β-actin as an internal control (**G**, **H**). Tests were separately performed for CDV:BS^M-P1^ (**A**, **C**, **E**, **H**) and CDV:BS-VP1^K98E,E145A,L169F^ (**B**, **D**, **F**, **G**). For **A**–**H**, a *t*-test with Welch's correction for unequal variance was used to compare mean values (*n*=4). Error bars represent the s.d.; *P<0.05, **P<0.005, ***P<0.0005, ****P<0.0001. clone-derived virus, CDV; multiplicity of infection, MOI; quantitative reverse transcription–PCR, qRT–PCR.

We performed the same assays described in the previous section to determine whether CDV:BS^M-P1^ and CDV:BS-VP1^K98E,E145A,L169F^ utilize mSCARB2 to infect murine cells. In viral uncoating experiments, we incubated the CDVs with soluble mSCARB2 protein for 2 h and quantified the total RNA extracted from the intact virus. Compared with untreated controls, incubation of CDV:BS^M-P1^ with 200 ng (*P*=0.0365) or 100 ng (*P*=0.0172) of soluble mSCARB2 significantly reduced the amount of intact viral RNA (Figure 7A). Similar results were observed in CDV:BS-VP1^K98E,E145A,L169F^ incubated with 200 ng (*P*=0.0013) or 100 ng (*P*=0.0352) of soluble mSCARB2 (Figure 7B). We also inoculated the mSCARB2-treated CDVs onto NIH/3T3 cells and observed that compared with untreated samples, cells inoculated with CDV:BS^M-P1^ treated with 25 ng (*P*=0.0349), 50 ng (*P*=0.0004), 100 ng (*P*<0.0001) or 200 ng (*P*<0.0001) of mSCARB2 contained lower amounts of viral RNA (Figure 7C). Similar results were observed in cells inoculated with CDV:BS-VP1^K98E,E145A,L169F^ pre-incubated with 100 ng (*P*=0.0114) or 200 ng (*P*=0.0003) of mSCARB2 (Figure 7D). The reduction in RNA was not observed in cells inoculated with virus pre-incubated with NSP.

In NIH/3T3 cells treated with anti-mSCARB2 rabbit serum prior to CDV inoculation, we observed that compared with mock-treated controls cells pre-incubated with low dilutions (1:20) of anti-mSCARB2 serum exhibited lower CDV:BS^M-P1^ titers (Figure 7E) but not CDV:BS-VP1^K98E,E145A,L169F^ titers (Figure 7F). Furthermore, cells pre-incubated with 2 μg (*P*=0.0017), 4 μg (*P*=0.0004) or 8 μg (*P*<0.0001) of anti-mSCARB2 serum prior to inoculation with CDV:BS^M-P1^ showed lower amounts of viral RNA compared with mock-incubated cells (Figure 7G). Similar results were obtained in cells pre-incubated with 2 μg (*P*=0.0015), 4 μg (*P*=0.0002) or 8 μg (*P*<0.0001) serum and inoculated with CDV:BS-VP1^K98E,E145A,L169F^ (Figure 7H). Together, these data confirm that CDV:BS^M-P1^ and CDV:BS-VP1^K98E,E145A,L169F^ recognize and interact with mSCARB2, which they use to infect murine cells.

### Infection phenotype induced by various EV71 strains in mice

**Figure 8:**
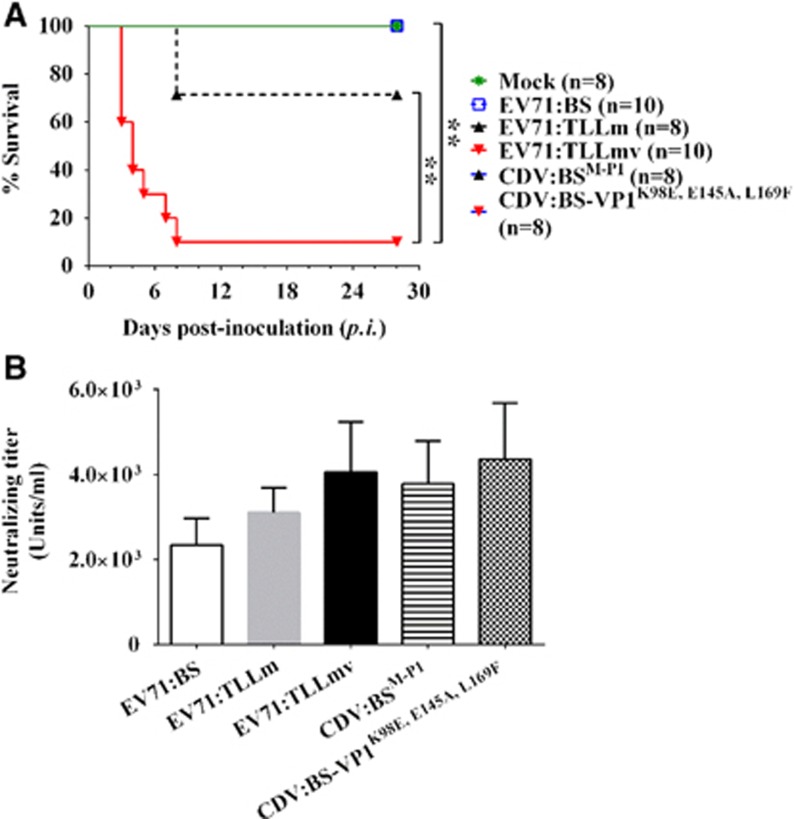
Inoculation of various EV71 strains and CDVs into BALB/c mice. (**A**) Kaplan–Meier survival curves of 6-day-old pups inoculated separately with EV71:BS, EV71:TTLLm, EV71:TLLmv, CDV:BS^M-P1^ or CDV:BS-VP1^K98E,E145A,L169F^. Mock-inoculated animals were injected with DMEM (1% FBS). (**B**) Neutralizing antibody titers in sera collected from inoculated pups that survived until the end of the 28-day observation period. For **A**, a log-rank (Mantel–Cox) test was used. For **B**, a *t*-test was used to compare mean values (*n*=8). Error bars represent the s.d. **P<0.005. clone-derived virus, CDV.

To determine whether various EV71 strains and CDVs can infect immunocompetent mice, we challenged 1-week-old BALB/c mice with 10^6^ CCID_50_ (median CCID) of virus via the i.p. route and observed the animals for 28 days. No significant signs of disease were observed in all mice inoculated with EV71:BS, CDV:BS-VP1^K98E,E145A,L169F^ or CDV:BS^M-P1^, and all mice survived throughout the study (Figure 8A). In contrast, EV71:TLLm and EV71:TLLmv induced significant morbidity that required euthanasia of the affected mice. At the end of the study, 70% of the pups inoculated with EV71:TLLm and 10% of those inoculated with EV71:TLLmv survived. Mice inoculated with EV71:TLLmv had a much shorter survival time compared with either mice inoculated with EV71:TLLm (*x*^*2*^=9.312; *P*=0.0023) or mock-infected controls (*x*^*2*^=7.648; *P*=0.0057) (Figure 8A). All inoculated animals exhibited seroconversion at the end of the observation period (Figure 8B) despite the absence of clinical disease in the majority of the animals. These results demonstrate that EV71:TLLm and EV71:TLLmv exhibit increased virulence in mice compared with the clinical isolate EV71:BS. Although CDV:BS^M-P1^ and CDV:BS-VP1^K98E,E145A,L169F^ productively infected murine cells, the mutations in their genomes are not sufficient to induce lethal disease in mice, suggesting that the molecular determinants for mouse virulence may reside in areas other than the capsid.

## DISCUSSION

In this study, we investigated the molecular determinants that enable the adapted strains EV71:TLLm and EV71:TLLmv to productively infect the mouse cell lines NIH/3T3 and Neuro-2a. First, we demonstrated that the restriction in EV71:BS infection of murine cells is at the stage of cellular entry and is due to the capsid protein. Replacement of the EV71:BS capsid with complementary sequences from EV71:TLLm enabled the resulting chimeric virus (CDV:BS^M-P1^) to productively infect NIH/3T3 and Neuro-2a cells. Second, we introduced individual substitutions into the VP1 (K98E, E145A and L169F) and VP2 (S144T and K149I) regions of the capsid protein by site-directed mutagenesis and determined which ones supported EV71:BS entry into murine cells by assaying the expression of viral proteins. All assayed CDVs were able to enter murine cells; however, only the VP1-L169F substitution permitted the resulting virus to produce viable progeny. We also generated infectious clones containing multiple substitutions in the VP1 and VP2 proteins and found that only CDV:BS-VP1^K98E,E145A^ and CDV:BS-VP1^K98E,E145A,L169F^ productively infected murine cells. These findings demonstrate that three VP1 residues (98E, 145A and 169F) are the minimum and necessary requirements for EV71:BS to productively infect murine cell lines. However, these substitutions are not sufficient to induce lethal disease in mice, suggesting that other determinants may govern virulence in mice.

As a prerequisite to cellular entry, the viral capsid must recognize and interact with the SCARB2 protein on host cells. SCARB2 interacts with the virus at the canyon region,^46, 50^ and this event leads to viral uncoating and entry of the genome into the cytoplasm. Our studies revealed that EV71:BS cannot enter murine cell lines predominantly due to a block in the cellular entry/viral uncoating process, and replacing the capsid protein with EV71:TLLm sequences reversed this. Moreover, introduction of at least three VP1 substitutions (K98E, E145A and L169F) into EV71:BS resulted in productive infection of murine cell lines, and the infection phenotype was sustainable for at least three viral passages in Neuro-2a cells. These experiments are similar to previous studies by Yamayoshi *et al.,*^22^ where they replaced exon 4 of the mouse SCARB2 (mSCARB2) gene with exon 4 of human SCARB2 (hSCARB2) in the L929 mouse cell line and demonstrated that this was sufficient to facilitate entry and replication of EV71 clinical isolates. These findings led us to hypothesize that EV71:TLLm and EV71:TLLmv could productively infect rodent cell lines because they have evolved to effectively utilize mSCARB2. To explore this possibility, we designed several viral entry/uncoating competitive assays and demonstrated that pre-incubation of the virus with soluble mSCARB2 protein reduced its cellular infectivity. Similarly, pre-incubation of NIH/3T3 cells with anti-mSCARB2 sera blocked viral infection. These experiments conclusively demonstrate that in contrast to EV71:BS, EV71:TLLm and EV71:TLLmv could utilize mSCARB2 to infect NIH/3T3 and Neuro-2a murine cell lines.

We also demonstrated that CDV:BS^M-P1^ and CDV:BS-VP1^K98E,E145A,L169F^ utilize mSCARB2 to infect murine cells. This led us to further hypothesize that the three VP1 substitutions may have been selected in the course of serial adaptation of EV71 to NIH/3T3 cells due to the improved interaction with mSCARB2 on murine cells. Circumstantial evidence indicates that this is a likely scenario. VP1-169 lies within the capsid canyon, and previous studies reported that mutations around the canyon of the poliovirus and rhinovirus capsids resulted in altered viral-binding kinetics with their receptors.^51, 52, 53^ It is also part of the SP55 peptide, which can elicit EV71-specific neutralizing antibodies.^47^ VP1-98 lies within a major immunodominant VP1 linear epitope that exhibits significant immunoreactivity with EV71-positive sera,^54^ and VP1-169 and VP1-145 reside within the region that contributes to the strength of VP1 self-association and interaction.^55^ The VP1-145E residue was also reported to increase cytotoxicity to Neuro-2a cells,^56^ while VP1-98 and VP1-145 are markers for viral binding to primate PSGL-1 proteins on leukocytes.^57, 58^ Nishimura *et al.*^57^ previously surveyed 1702 VP1 protein sequences from EV71 clinical isolates published in GenBank. Analyzing this database of sequences, which can be found in the Supplementary Materials and Methods,^57^ we observed that the amino acids VP1-98E and 145A appeared in the same genome in only 5 out of 1702 isolates (0.3%), and VP1-169F was not observed in the database at all. These observations suggest that the amino acid combination of VP1-98E, 145A and 169F is novel and has not been identified in any EV71 isolate, supporting our theory of the relevance of these three substitutions during the adaptation process in NIH/3T3 cells. It would be of scientific interest to test these five strains (GenBank Accession NO AB059814, AB524121, AF135940, HQ667783 and HQ667786) for their ability to productively infect NIH/3T3 and Neuro-2a cell lines, especially following introduction of the 169F substitution.

In an effort to provide further support for our hypothesis that the three VP1 substitutions enabled EV71 to more efficiently interact with mSCARB2, we performed pull-down assays on CDV:BS-VP1^K98E,E145A,L169F^ using agarose beads coated with recombinant soluble mSCARB2 proteins. However, this experiment to demonstrate sustained binding of the virus to mSCARB2 was not successful despite multiple attempts. In similar control experiments, the mSCARB2-coated beads could not pull down EV71:TLLm or EV71:TLLmv (Supplementary Figure S8), suggesting that perhaps the interaction between mSCARB2 and these viruses is transient. Despite this, we showed in this study that incubation of either an adapted virus or CDV:BS-VP1^K98E,E145A,L169F^ with mSCARB2 leads to viral uncoating. This is a crucial point because other cellular proteins have previously been reported to interact with or bind to EV71^43, 59, 60, 61, 62, 63, 64^ (either specifically or non-specifically), but only SCARB2 has so far been shown to induce viral uncoating.^19, 50^

This is the first study that successfully identified specific amino acid substitutions in the capsid that enabled EV71 to productively infect non-susceptible mouse cell lines, specifically NIH/3T3 and Neuro-2a. Identifying the mutations required for EV71 to productively infect murine cells could provide information on the interactions with mSCARB2 that are necessary for the virus to ‘evolve' or develop to utilize the specific receptor. We also emphasize that EV71:TLLm and EV71:TLLmv are the first reported EV71 strains that successfully utilize the physiologically expressed mSCARB2 protein to infect murine cells. Previous attempts to enable EV71 infection of murine cells relied on ectopic expression of the hSCARB2 protein. Although we still do not fully understand why or how these three VP1 residues enable productive infection of murine cells, the recently solved three-dimensional structure of hSCARB2 may reveal the underlying mechanisms of why EV71 cannot utilize mSCARB2 to infect murine cells, and consequently provide clues for how the combination of VP1-98E, 145 and 169F enable EV71 to utilize mSCARB2. A large cavity or tunnel, which can accommodate sphingosine and other lipid molecules, traverses the length of the protein,^23^ and access to this tunnel is regulated by a cap structure that maintains the tunnel in a ‘closed conformation' at neutral pH and an ‘open conformation' at acidic pH.^50^ Interestingly, this cap structure is formed by part of exon 4 (aa 142–164) of the protein, which is known to be highly divergent between mSCARB2 and hSCARB2 and is important for EV71 binding and cellular infection.^22^ Furthermore, in this proposed model, protonation of His150 of hSCARB2 at an acidic pH within the endosome triggers the structural rearrangement required to open the lipid tunnel, which subsequently sequesters the sphingosine displaced from the virus canyon. In molecular homology models of the mSCARB2 protein, His150 is replaced by threonine, and the tunnel interior is more hydrophobic compared with hSCARB2. This suggests that acidification is not sufficient to open the lipid tunnel, and another trigger—perhaps a specific interaction with the virus—may be required. Further investigations are necessary to explore this scenario in mSCARB2-dependent EV71 infection of murine cells, and our adapted strains provide opportunities for potential future collaborations to address these unsolved questions.
