# Supplementary material for: Cooperative effect of the VP1 amino acids 98E, 145A and 169F in the productive infection of mouse cell lines by enterovirus 71 (BS strain)
Source: Emerg Microbes Infect. 2016 Jun 22;5(6):e60–. doi: 10.1038/emi.2016.56 (PMC4932649; doi:10.1038/emi.2016.56)
Supplement: Supplementary Table 1 [file emi201656x1.pdf]

Supplementary Table S1

List of primers used for the production of infectious cDNA clones

Primer name	Sequence
**BamHI-PfF**	5' CTA GGG ATC C[1]TA ATA CGA CTC ACT ATA[2] GGTTCA AAC AGC CTGTGG GTT GCA CCC ACT CAC AGG 3'
**Pf-AatIIR**	5' CTA GGA CGT C[3]CG GCC G[4]AA CTT TCC AAG GGT AGT AAT GGC AGT ACG ACT AGT GCC 3'
**HindIII-DF**	5' TAA TAA GCT T[5]CG GCC G[4] GC AGT CTG GGG CCA TCT ACG TG 3'
**D-BamHIR**	5' GCG CGG ATC C[1]TT TTT TTT TTT TTT TTT TTT GCT ATT CTG GTT ATA ACA AAT TTA CCC CCA C 3'
**SDM_MluIF**	5' GGT GTC CAC TCA ACG CGT[6] CGG CTC CCA CGA GAA CTC CAA TTC AGC TAC AGA AGG CTC C 3'
**SDM_MluIR**	5' CGT GGG AGC CGA CGC G T[6]T GAG TGG ACA CCT GTG AGC CCA TGC TTG 3'
**MluI-TLLm-P1F**	5' ACT CAA CGC GT[6]C GGC TCC CAC GAG AAC TCC AAT TCA GCT ACA GAA GGC 3'
**EagI-TLLm-P1R**	5' ACT GCC GGC CG[4]A ACT TTC CAA GGG TAG TAA TGG CAG TAC GAC TAG TGC C 3'
**VP2-G1385C-F**	5' CAG AGG ACA ***C***CC ACC CTC CTT ACA AAC AAA CAC AAC CTG GCG CC 3'
**VP2-G1385C-R**	5' GGA GGG TGG ***G***TG TCC TCT GTT CCT GTA CCG CCT G 3'
**VP2-A1400T-F**	5' CTCCTTACA***T***ACAAACACAACCTGGCGCCGACG 3'
**VP2-A1400T-R**	5' TGTGTTTGT***A***TGTAAGGAGGGTGGCTGTCCTCTGTTCC 3'
**VP1-A2734G-F**	5' CTCCCTCTT***G***AGGGTACCACCAATCCAAATGGTTATGCCAACTGGG 3'
**VP1-A2734G-F**	5' TGGTACCCT**C**AAGAGGGAGATCTATCTCTCCTACCAAACCTGCCC 3'
**VP1-A2876C-F**	5' CTACTGGTG***C***GGTTGTTCCACAATTACTCCAGTATATGTTTGTTCCCCC TGG 3'
**VP1-A2876C-R**	5' GGAACAACC***G***CACCAGTAGGAGTGCACGCAACAAAAGTGAATT 3'
**VP1-C2947T-F**	5' AGAGAATCA***T***TTGCTTGGCAGACAGCCACAAACCCC 3'
**VP1-C2947T-R**	5' GCCAAGCAA***A***TGATTCTCTAGACTCTGGTTTGGGAGCACC 3'

[1] BamHI recognition site
[2] The T7 polymerase recognition site is underlined. The sequence is followed by GG prior to the beginning of EV71 5'UTR
[3] AatII recognition site
[4] EagI recognition site
[5] HindIII recognition site
[6] MluI recognition site
