# Supplementary material for: Cooperative effect of the VP1 amino acids 98E, 145A and 169F in the productive infection of mouse cell lines by enterovirus 71 (BS strain)
Source: Emerg Microbes Infect. 2016 Jun 22;5(6):e60–. doi: 10.1038/emi.2016.56 (PMC4932649; doi:10.1038/emi.2016.56)
Supplement: Supplementary Table 2 [file emi201656x2.pdf]

Supplementary Table S2

List of all constructed plasmid clones and resulting clone-derived virus (CDV)

Plasmid name	Detailed information
**pACYC-BSPf**	Proximal fragment containing the 5'UTR and P1 sequences of EV71:BS and cloned into pACYC177
**pACYC-BSD**	Distal fragment containing the P2, P3, and 3'UTR sequences of EV71:BS and cloned into pACYC177
**pACYC-BS**	Proximal and distal fragments encoding the full-length genomic cDNA of EV71:BS cloned into pACYC177.<br>The resulting CDV is written as CDV:BS
**pACYC-BS$^{\Delta M\text{-}P1}$**	Plasmid where the capsid-encoding (P1) region of pACYC-BS is replaced with equivalent sequences from EV71:TLLm.<br>The resulting CDV is written as CDV:BS$^{M\text{-}P1}$
**pACYC-BSPf$^{\Delta M\text{-}P1}$**	Plasmid containing the proximal fragment of EV71:BS, where the P1 region is replaced with equivalent sequences from EV71:TLLm
**pBS-VP2^{S144T}**	pACYC-BS mutagenized to introduce an S → T amino acid substitution in VP2-144 residue using the following primers: VP2-G1385C-F and VP2-G1385C-R.<br>The primers introduce a G→C base change at nucleotide position 1385.<br>The resulting CDV is written as CDV:BS-VP2^{S144T}
**pBS-VP2^{K149I}**	pACYC-BS mutagenized to introduce a K → I amino acid substitution in VP2-149 residue using the following primers: VP2-A1400T-F and VP2-A1400T-R.<br>The primers introduce an A→T base change at nucleotide position 1400.<br>The resulting CDV is written as CDV:BS-VP2^{K149I}
**pBS-VP1^{K98E}**	pACYC-BS mutagenized to introduce a K → E amino acid substitution in VP1-98 residue using the following primers: VP1-A2734G-F and VP1-A2734G-R.<br>The primers introduce an A→G base change at nucleotide position 2734.<br>The resulting CDV is written as CDV:BS-VP1^{K98E}
**pBS-VP1^{E145A}**	pACYC-BS mutagenized to introduce an E → A amino acid substitution in VP1-145 residue using the following primers: VP1-A2876C-F and VP1-A2876C-R.<br>The primers introduce an A→C base change at nucleotide position 2876.<br>The resulting CDV is written as CDV:BS-VP1^{E145A}
**pBS-VP1^{L169F}**	pACYC-BS mutagenized to introduce an L → F amino acid substitution in VP1-169 residue using the following primers: VP1-C2947T-F and VP1-C2947T-R.<br>The primers introduce an C→T base change at nucleotide position 2947.<br>The resulting CDV is written as CDV:BS-VP1^{L169F}
**pBS-VP2$^{S144T, K149I}$**	pACYC-BS mutagenized to introduce two amino acid substitutions at VP2: 144$^{Ser\rightarrow Thr}$ and 149$^{Lys\rightarrow Ile}$<br>The resulting CDV is written as CDV:BS-VP2$^{S144T, K149I}$
**pBS-VP1$^{K98E, E145A}$**	pACYC-BS mutagenized to introduce two amino acid substitutions at VP1: 98$^{Lys\rightarrow Glu}$ and 145$^{Glu\rightarrow Ala}$<br>The resulting CDV is written as CDV:BS-VP1$^{K98E, E145A}$

**pBS-VP1$^{K98E, E145A, L169F}$**	pACYC-BS mutagenized to introduce three amino acid substitutions at VP1: $98^{Lys \rightarrow Glu}$, $145^{Glu \rightarrow Ala}$ and $169^{Leu \rightarrow Phe}$<br>The resulting CDV is written as CDV:BS-VP1$^{K98E, E145A, L169F}$
**pBS-VP1, VP2**	pACYC-BS mutagenized to introduce two amino acid substitutions at VP2: $144^{Ser \rightarrow Thr}$ and $149^{Lys \rightarrow Ile}$; and three amino acid substitutions at VP1: $98^{Lys \rightarrow Glu}$, $145^{Glu \rightarrow Ala}$ and $169^{Leu \rightarrow Phe}$<br>The resulting CDV is written as CDV:BS-VP1, VP2
