# Supplementary material for: Cooperative effect of the VP1 amino acids 98E, 145A and 169F in the productive infection of mouse cell lines by enterovirus 71 (BS strain)
Source: Emerg Microbes Infect. 2016 Jun 22;5(6):e60–. doi: 10.1038/emi.2016.56 (PMC4932649; doi:10.1038/emi.2016.56)
Supplement: Supplementary Table 3 [file emi201656x3.pdf]

Supplementary Table S3

List of primers and probes used in quantitative RT-PCR

Primer name	Sequence information
**EV5’NC-F**	5’ AATAAATCATAACCTCCGGCCCCTGAATG 3’
**EV5’NC-R**	5’ AATAAATCATAAGAAACACGGACACCCAAAGTAGTC 3’
**EV5’NC Taqman probe**	5’ [6FAM][7] TCCGCTGCAGAGTTRCCCGTTACGA [TAMRA][8] 3’
**β-actin-F**	5’ AATAAATCATAACCBTCCTTCYTGGGY 3’
**β-actin-R**	5’ AATAAATCATAAGAGGAGCRATGATCT 3’
**β-actin Taqman probe**	5’ [HEX][7] TCCATCATGAAGTGYGACGTBGACATCCG [TAMRA][9] 3’

[7] 6FAM (6-carboxyfluorescein)
[8] TAMRA (tetramethylrhodamine)
[9] HEX (hexachlorofluorescein)
