# Supplementary material for: Cooperative effect of the VP1 amino acids 98E, 145A and 169F in the productive infection of mouse cell lines by enterovirus 71 (BS strain)
Source: Emerg Microbes Infect. 2016 Jun 22;5(6):e60–. doi: 10.1038/emi.2016.56 (PMC4932649; doi:10.1038/emi.2016.56)
Supplement: Supplementary Information [file emi201656x4.pdf]

SUPPLEMENTARY MATERIALS and METHODS

Construction of EV71:BS full-length cDNA clones. All plasmid amplifications were done by transforming synthesized constructs into The XL-10 Gold ultracompetent *E. coli* strain (Stratagene, USA). Full-length EV71:BS cDNA clones were constructed by a two-step cloning procedure (**Supplementary Figure S1B**). Two fragments - proximal (BSPf) and distal (BSD), were independently cloned into plasmid pACYC177 (New England Biolabs, Singapore). The proximal fragment contains the 5'UTR (untranslated region) and P1 regions of the viral genome, while the distal fragment contains the P2, P3, and 3'UTR regions. The BSPf fragment was amplified using the primer pair BamHI-PfF and Pf-AatIIR, and subsequently digested with BamHI and AatII endonucleases. The recipient plasmid pACYC177 was also digested with the same enzyme pair, and the two fragments were ligated together to form the pACYC-BSPf construct. Similarly, the BSD fragment was amplified using the primer pair HindIII-DF and D-BamHIR. The BSD fragment and pACYC177 plasmid were simultaneously digested with HindIII and BamHI restriction enzymes and subsequently ligated together to produce the pACYC-BSD construct.

The BamHI-PfF primer includes a sequence of the T7 polymerase promoter region upstream of the 5'UTR (**Supplementary Table S1**) to facilitate transcription with T7 polymerase enzyme, which is ectopically expressed from another plasmid (pCMV-T7Pol). The Pf-AatIIR and HindIII-DF primers also include an internal recognition site of EagI endonuclease (5'-CGGCCG-3') for subsequent excision and replacement of the P1 region. To produce EV71:BS full-length cDNA plasmid pACYC-BS, pACYC-BSPf and pACYC-BSD were digested with EagI and AatII endonucleases, gel-purified, and ligated together.

Construction of chimeric EV71:BS plasmid encoding EV71:TLLm capsid protein sequences. Chimeric pACYC-BS$^{\Delta M\text{-}P1}$ cDNA clones, where the capsid region of pACYC-BS was replaced with EV71:TLLm capsid sequences, were produced by standard cloning procedures (**Supplementary Figure S1C**). We engineered a recognition site for MluI endonuclease (5'-ACGCGT-3') within the

pACYC-BSPf plasmid via standard site-directed mutagenesis[30] using the primer pair SDM_MluIF and SDM_MluIR. The P1 region of EV71:TLLm gene was amplified using the primer pair MluI-TLLm-P1F and EagI-TLLm-P1R. The resulting TLLm-P1 PCR amplicon and pACYC-BSPf plasmid were digested with MluI and EagI endonucleases, and subsequently ligated to produce pACYC-BSPf$^{\Delta M\text{-}P1}$. The full-length cDNA clone (pACYC-BS$^{\Delta M\text{-}P1}$) was generated by digesting pACYC-BSD and pACYC-BSPf$^{\Delta M\text{-}P1}$ with AatII and EagI endonucleases. The fragments were subsequently purified and ligated together.

Construction of mutant plasmid EV71:BS cDNA clones with amino acid substitutions in VP1 and VP2 proteins. The plasmid pACYC-BSPf was subjected to site-directed mutagenesis using relevant primer pairs (see **Supplementary Table S1**) to introduce specific mutations into the VP1 and/or VP2 gene sequences (**Figure 2A, 3A**). The mutagenized plasmids were subsequently ligated with pACYC-BSD as described above. The resulting mutated plasmid clones are all listed in **Supplementary Table S2.**

Expression and purification of recombinant soluble SCARB2 proteins. The plasmids encoding murine Scavenger Receptor Class B Member-2 (SCARB2) cDNA (pMD18-mSCARB2; Genbank accession no. NP_031670.1) and human SCARB2 cDNA (pMB18-hSCARB2; accession no. NM_007644.3) were purchased from Sino Biological, Inc. (Beijing, China). The pQE30 vector (Qiagen, Germany) was used in recombinant expression of soluble SCARB2 proteins in *E. coli*. The *E. coli* BL21 strain (New England Biolabs, UK) was used in recombinant expression of SCARB2 proteins.

The plasmids pMD18-mSCARB2 and pMD18-hSCARB2 encoding the extracellular domains of SCARB2 protein (aa Arg27-Thr432) were amplified and cloned into the pQE30 protein expression vector. The clones were transformed into BL21 *E. coli* cells, and protein expression was induced with 1mM IPTG for 12-16h at 25° C. Proteins were subsequently collected by digesting the harvested cell pellets with lysozyme (1mg/ml), and the crude extract was purified in Ni-NTA column (Qiagen,

Germany). Briefly, cleared lysates were incubated overnight in 50% Ni-NTA slurry (4:1 ratio) at 4° C with gentle shaking and passed through the columns. The beads were washed 5x in wash buffer (50mM NaH_2PO_4, 300mM NaCl, 20mM imidazole; pH 8.0), and proteins were eluted with elution buffer (50mM NaH_2PO_4, 300mM NaCl, 250mM imidazole; pH 8.0). Purified proteins were quantified by incubation with the Bradford Assay reagent (Bio-Rad, USA) for 15min RT and measuring the absorbance at 595nm. Samples were stored in -20° C until further use.

Production of rabbit antisera to mouse SCARB2 proteins. The procedures for production of rabbit polyclonal sera were approved by the Temasek Lifesciences Laboratory Institution Animal Care and Use Committee (TLL-IACUC) [Approval No. 047/12]. Two healthy male rabbits were immunized with 1.4µg purified mSCARB2 protein mixed with Freund's complete adjuvant at day 0. Booster containing 0.8µg antigen mixed with Freund's incomplete adjuvant was injected at days 21, 42, 63, 84, and 105. Blood was terminally collected by cardiac puncture at day 117, and the collected blood was incubated overnight at 4° C prior to centrifugation at 3,000rpm for 30min. Cleared serum was collected and purified using Hi-Trap Protein G columns (GE Healthcare, USA) following the manufacturer's recommended protocols. Sera were stored at -20° C until further use.

Measurement of neutralizing antibody levels in sera from infected mice. Blood samples were collected by cardiac puncture at necropsy before being clotted at room temperature. Sera were obtained by centrifugation 3000 *x*g, 20min, 4°C. Samples were stored at -20° C until further analysis.
Random samples of the frozen stock sera were assayed for neutralizing antibody titers. Two-fold serial dilutions of serum (1:20 to 1:1280) were prepared in 96-well plates and mixed with 100 CCID50 virus or CDV. The mixture was incubated for 1h at 37° C prior to addition of Vero cells (6,000 cells/ well). Plates were incubated at 37° C for several days and observed for CPE between days 4-10. Neutralizing antibody titers were determined using the Reed and Muench method (reported as units per ml sera).

Western blots and pull-down assays. To determine the specificity of anti-mSCARB2 rabbit sera, 1µg purified mSCARB2 and hSCARB2 were resolved in 14% native polyacrylamide gels in native running buffer (25mM Tris, 192mM glycine; pH 8.3). Proteins were blotted onto PVDF membranes by wet transfer (90V, 2h, 4° C) and blocked with 5% non-fat milk (Bio-Rad, USA) in PBS-T (PBS, pH 7.4; 0.1% Tween-20) for 1h, RT. Membranes were probed with anti-mSCARB2 rabbit serum (1:2000 dilution; 5% milk, PBS-T) overnight at 4° C and washed 5x with PBS-T at 5min intervals. Membranes were incubated with swine anti-rabbit Ig-HRP (Dako Cytomation, Denmark; 1:5000 dilution; 1% milk, PBS-T) for 2h, RT and subsequently washed 5x with PBS-T at 5min intervals. Signals were detected with Clarity™ Western ECL substrate and ChemiDoc™ imaging system (Bio-Rad, USA).

To determine if the anti-mSCARB2 rabbit sera recognize native cellular SCARB2 proteins, overnight seeded Vero, NIH/3T3, and Neuro-2a cells were washed with cold PBS 3x and harvested in RIPA buffer (20mM Tris-HCl, pH 7.5; 150mM NaCl; 1mM Na_2EDTA; 1mM EGTA; 1% NP-40; 1% sodium deoxycholate) supplemented with 1x Complete protease inhibitor cocktail (Roche, USA). 40µg of protein samples were resolved in 14% denaturing polyacrylamide gels and blotted onto PVDF membranes by wet transfer. Proteins were probed similarly as already described.

For pull-down assays, Neuro-2a cells were inoculated with 10 MOI virus (EV71:BS, EV71:TLLm or EV71:TLLmv) for 1h 4° C and washed 3x with cold PBS. Proteins were collected with RIPA buffer and quantified as already described. Protein A/G conjugated agarose beads were incubated overnight with anti-EV71 guinea pig sera (1:10 dilution) that recognizes native EV71 virus, washed with cold PBS 3x, and mixed with 100µg protein lysate (1:1) for 3h at 4° C. Agarose beads were washed 5x with cold PBS and subsequently boiled for 5min in treatment buffer (62.5mM Tri-HCl, pH 6.8; 2.5% SDS; 0.002% bromophenol blue; 5% β-mercaptoethanol; 10% glycerol) prior to loading onto 14% denaturing polyacrylamide gels. After blotting the proteins onto PVDF membranes by wet transfer, SCARB2 proteins were probed with anti-SCARB2 mouse polyclonal sera (1:2000) and detected as already described.
