# Supplementary figures and images for: Cooperative effect of the VP1 amino acids 98E, 145A and 169F in the productive infection of mouse cell lines by enterovirus 71 (BS strain)
Source: Emerg Microbes Infect. 2016 Jun 22;5(6):e60–. doi: 10.1038/emi.2016.56 (PMC4932649; doi:10.1038/emi.2016.56)

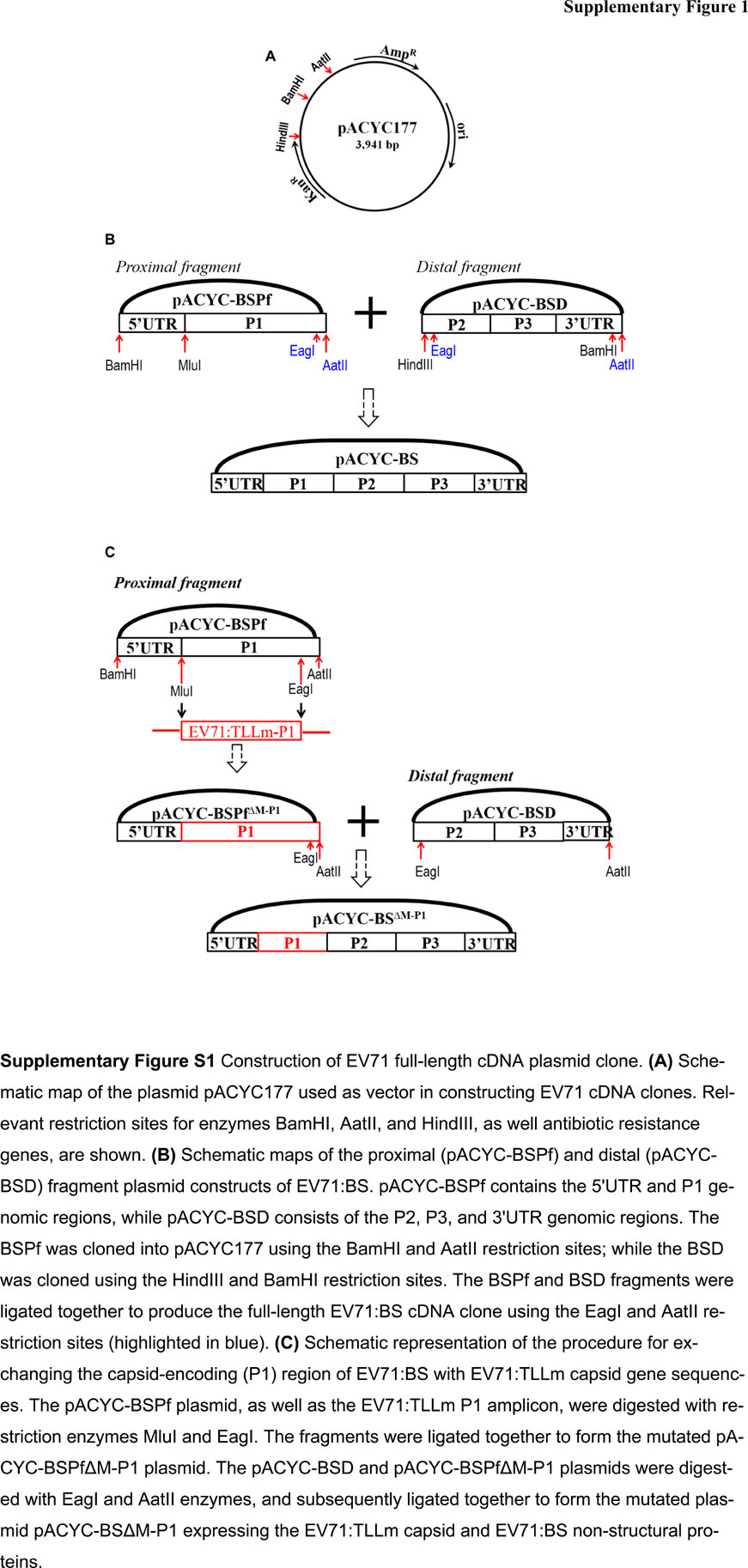

Supplement: Supplementary Figure S1 [file emi201656x5.tif]

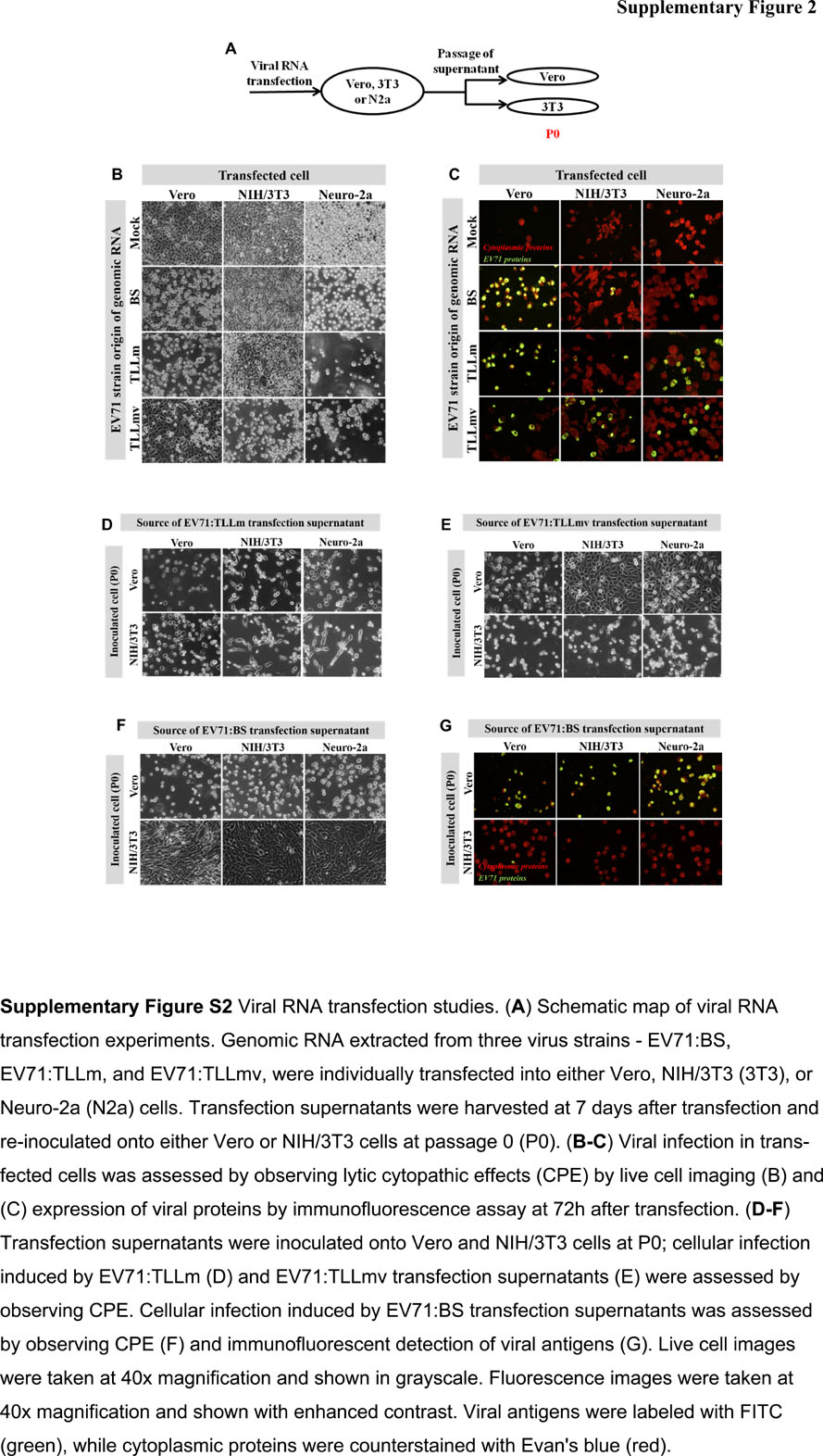

Supplement: Supplementary Figure S2 [file emi201656x6.tif]

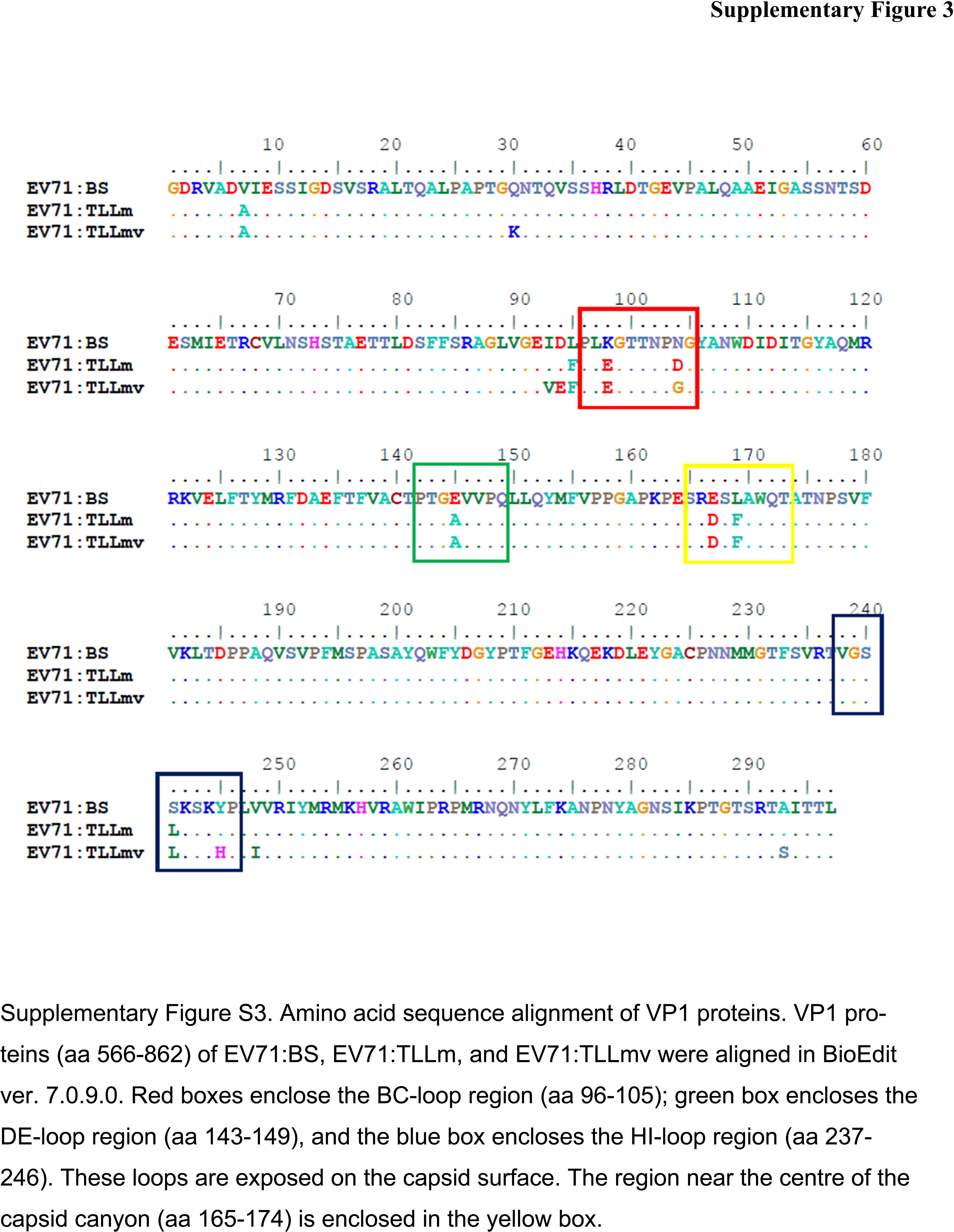

Supplement: Supplementary Figure S3 [file emi201656x7.tif]

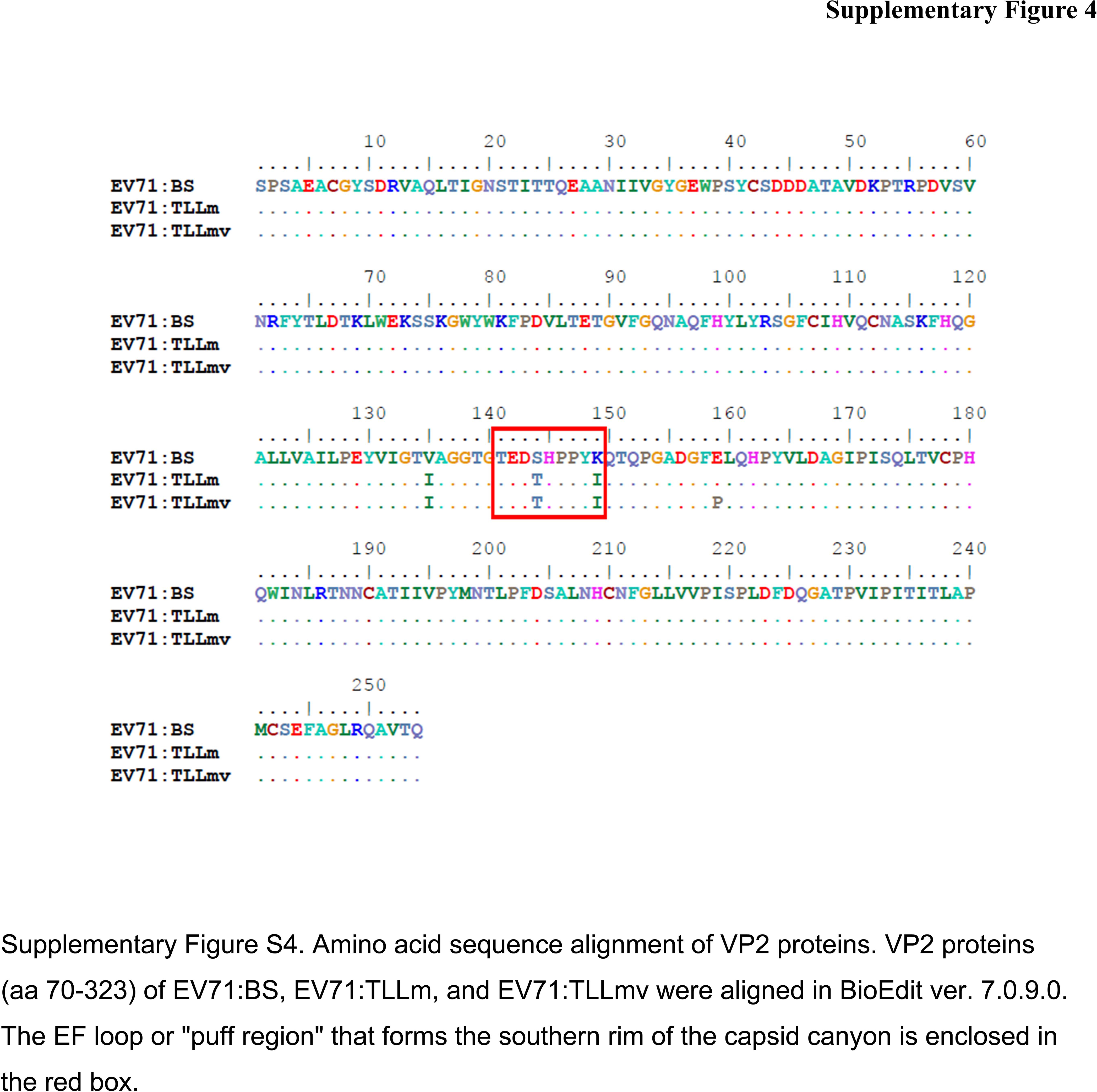

Supplement: Supplementary Figure S4 [file emi201656x8.tif]

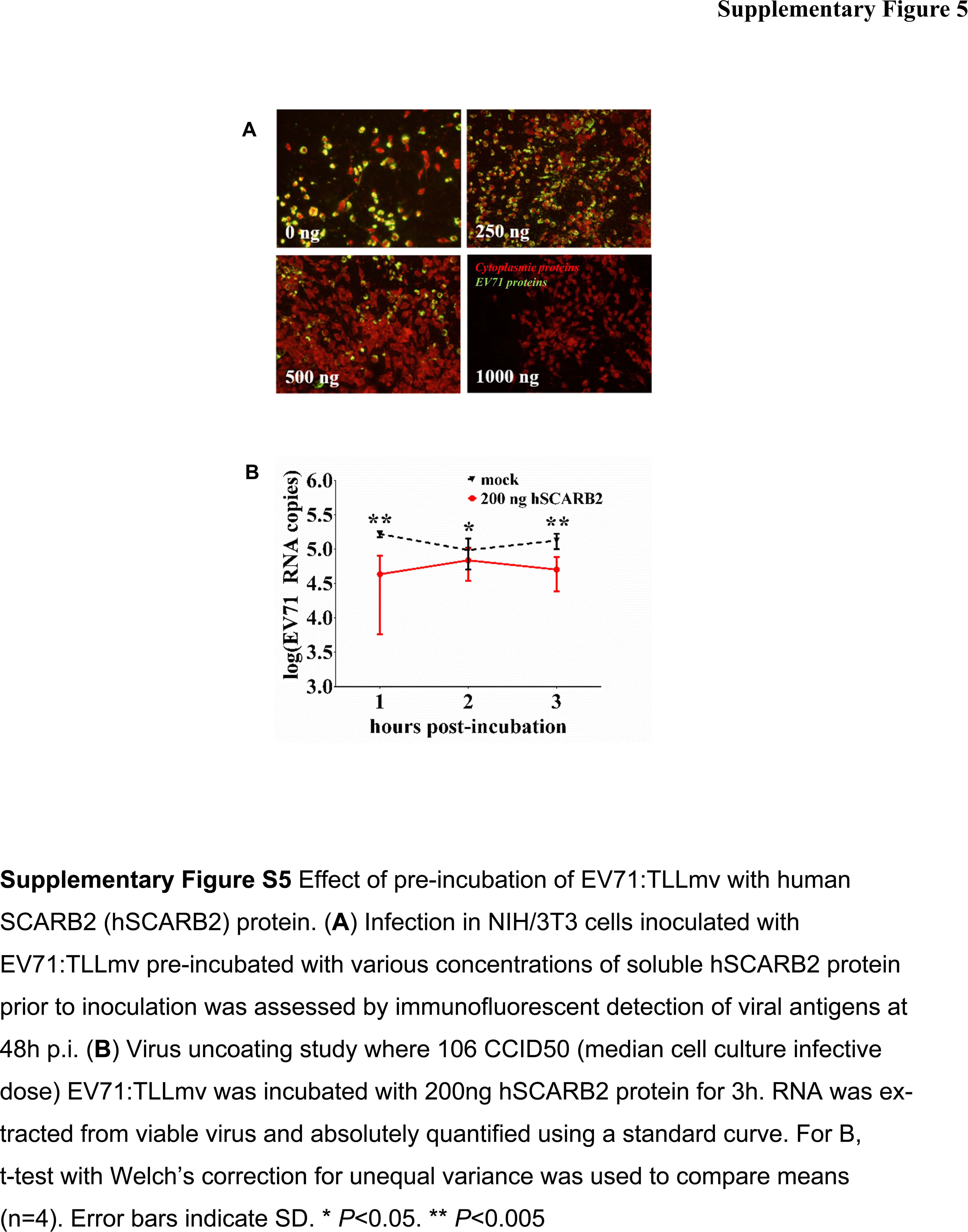

Supplement: Supplementary Figure S5 [file emi201656x9.tif]

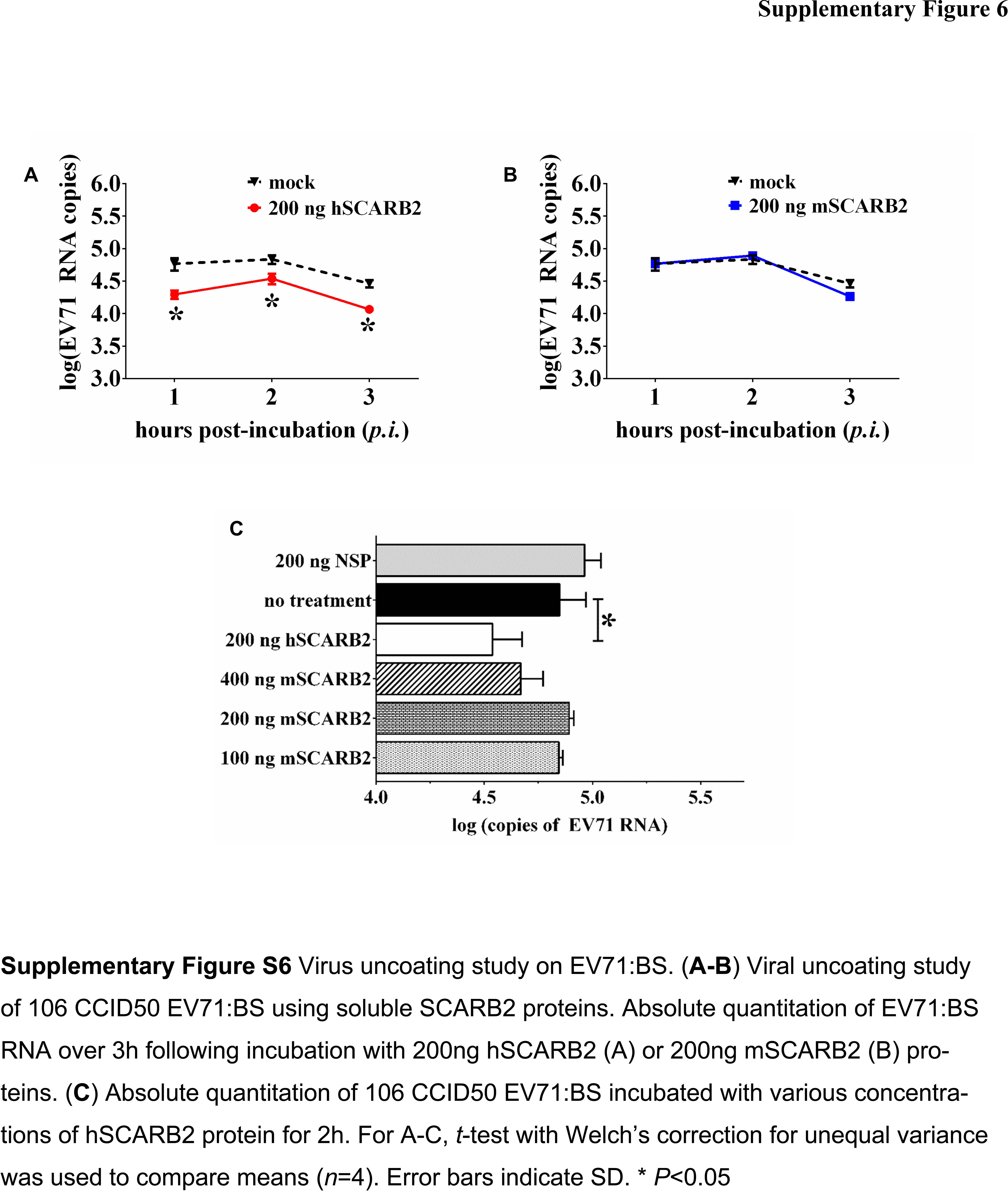

Supplement: Supplementary Figure S6 [file emi201656x10.tif]

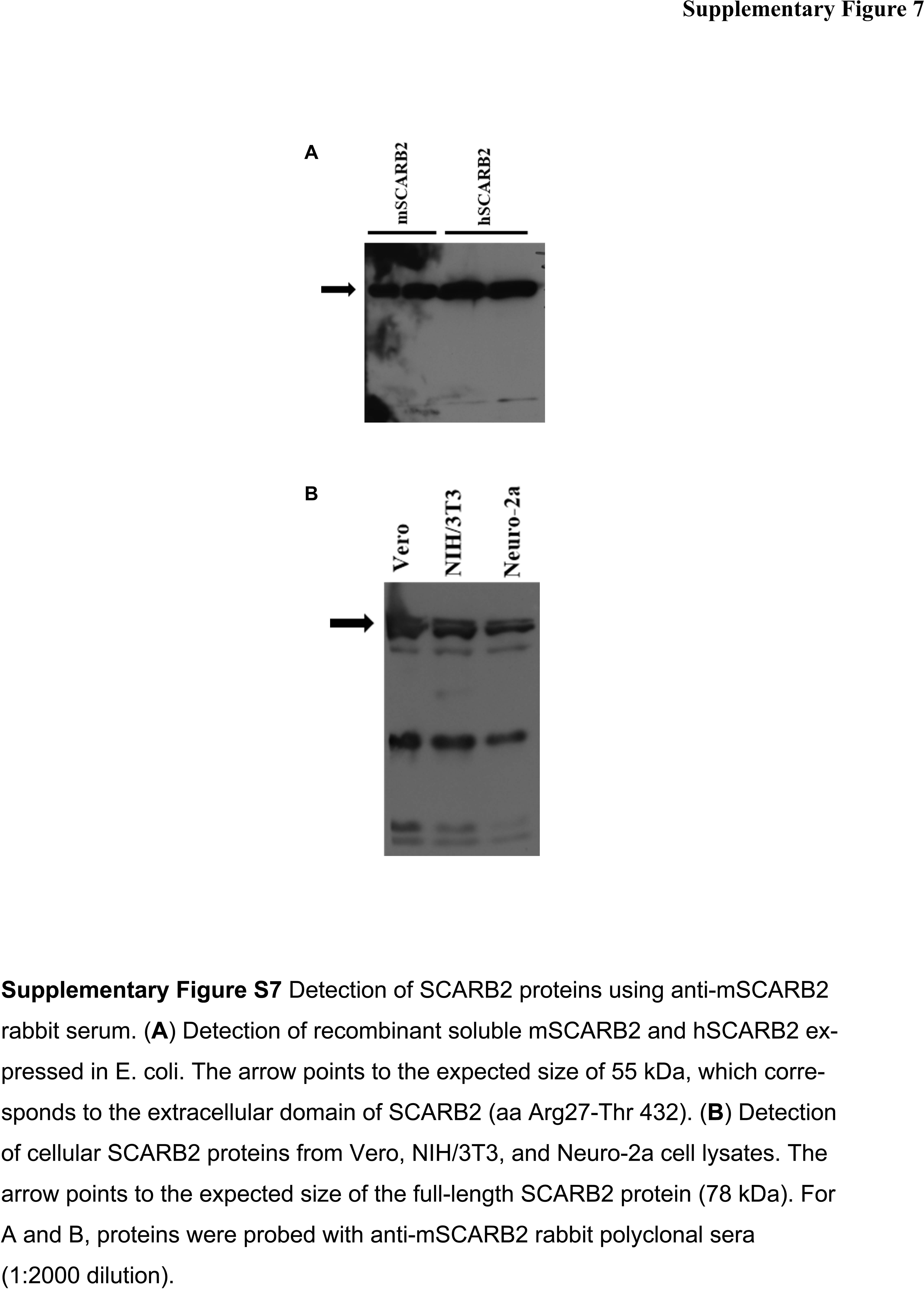

Supplement: Supplementary Figure S7 [file emi201656x11.tif]

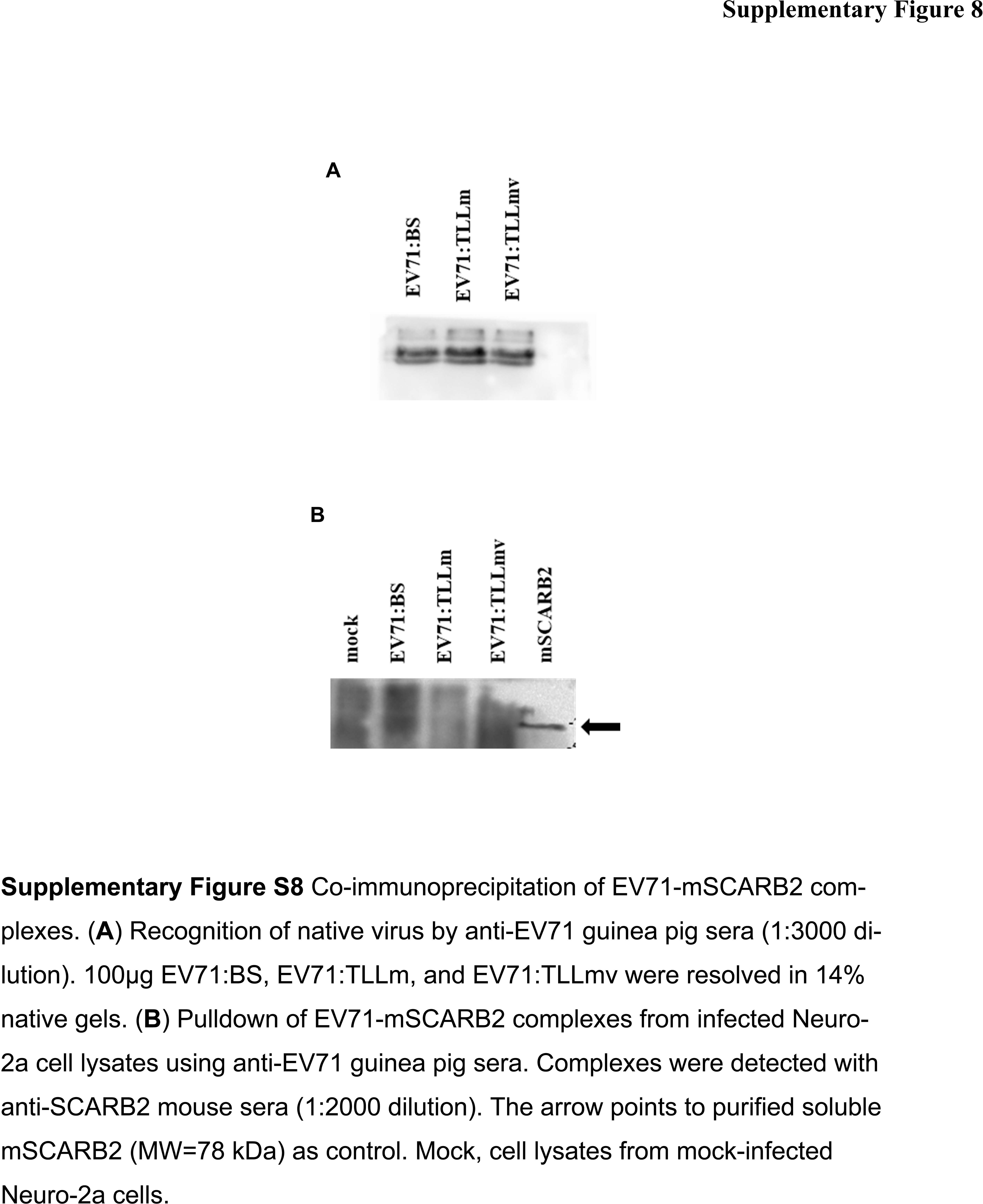

Supplement: Supplementary Figure S8 [file emi201656x12.tif]
